# Spatiotemporal epidemiology and hierarchical analysis of suicide mortality and associated risk factors in Thailand using national surveillance data during 1997–2021

**DOI:** 10.1038/s41598-025-20481-0

**Published:** 2025-10-09

**Authors:** Chawarat Rotejanaprasert, Chiraphat Phoncharoenwirot, Papin Thanutchapat, Ornrakorn Mekchaiporn, Peerut Chienwichai, Terdsak Detkong, Richard James Maude

**Affiliations:** 1 Department of Tropical Hygiene Faculty of Tropical Medicine , Mahidol University , Bangkok, Thailand; 2 Mahidol-Oxford Tropical Medicine Research Unit, Faculty of Tropical Medicine , Mahidol University , Bangkok, Thailand; 3 Princess Srisavangavadhana College of Medicine , Chulabhorn Royal Academy , Bangkok, Thailand; 4 Department of Computer Engineering, Faculty of Engineering, King Mongkut’s University of technology Thonburi, Bangkok, Thailand; 5 Department of Mental Health, Ministry of Public Health , Nonthaburi, Thailand; 6 Nuffield Department of Medicine, Centre for Tropical Medicine and Global Health, University of Oxford , Oxford, UK; 7 The Open University , Milton Keynes, UK

**Keywords:** Spatiotemporal, Suicide, Thailand, Mental health, Policy, Hierarchical modeling, Public health, Psychiatric disorders

## Abstract

Suicide as a critical but neglected public health concern in Asia. In Thailand, limited research exists on suicide, further complicated by varying rates across regions and sex disparities. In this context, understanding the nuanced spatiotemporal dimensions of suicide not only illuminates its intricate dynamics but can also guide targeted interventions and more effective mental health policies. Addressing this gap, our study conducted an extensive analysis of routine Thai suicide report data spanning 1997 to 2021 from the Department of Mental Health, Thai Ministry of Public Health. Socioeconomic indicators were sourced from the National Statistical Office of Thailand. Bayesian spatiotemporal hierarchical models were employed to investigate suicide trends and associations with risk factors. Suicide cluster detection was conducted to identify high-risk areas. Between 1997 and 2021, Thailand recorded 106,955 suicide deaths, with males accounting for 78% of the total. The national suicide rate peaked in 1999 and subsequently declined by 29.4% through 2006, followed by a moderate increase of 11.5% from 2018 to 2021. The northern region consistently exhibited the highest crude suicide rates across the study period. A persistent sex disparity was observed: male suicide rates were more than three times higher than female rates and showed greater temporal variability. Suicide hotspots were identified primarily in northern and eastern provinces. Significant associations were found between suicide risk and financial distress as well as crime-related indicators, reflecting complex spatial and temporal dynamics. This research marks a significant stride in unraveling the spatiotemporal dynamics of suicide in Thailand. The distinctive regional and sex-specific trends highlight the need for interventions responsive to these nuances while embracing a holistic perspective. By emphasizing the integration of spatiotemporal insights into policy frameworks, this research offers valuable guidance for crafting targeted mental health strategies, ultimately fostering enhanced well-being throughout Thailand.

**Supplementary Information:**

The online version contains supplementary material available at 10.1038/s41598-025-20481-0.

## Introduction

Suicide is a critical global public health issue, especially in low- and middle-income countries, where it accounts for a substantial proportion of premature mortality. An estimated 40% of all suicides occur in Asia^1^, with several countries in the region—such as Japan, India, and Thailand—reporting persistently high suicide rates. Japan, for example, has historically experienced some of the highest suicide rates among G7 nations, with over 30,000 deaths annually between 1998 and 2011, followed by a decline attributed to intensified suicide prevention policies^2,3^. In India, southern states consistently report higher suicide rates than northern regions, likely due to both underlying socioeconomic factors and more complete death registration systems^4^.

Thailand, situated in Southeast Asia, also faces a significant suicide burden^5^. As of 2019, Thailand reported 8 suicide deaths per 100,000 population, compared to 5.5 in Cambodia and 2.5 in the Philippines^6^. In 2020, this rate had declined slightly to 7.8 per 100,000 population^5^. Between 2013 and 2019, approximately 80% of suicide deaths in Thailand involved men, with an average age of 45.4 years, and national trends indicate a gradual increase in suicide incidence during this period^7^. Estimates suggest that a suicide attempt occurs every 10 min in Thailand^6^. In 2018, the national suicide rate stood at 6.3 per 100,000 population, with the majority of deaths occurring among working-age individuals, reflecting the broader socioeconomic costs of suicide^8^.

Despite these figures, suicide epidemiology in Thailand remains poorly understood, particularly at the subnational level. Prior Thai studies have identified factors such as alcohol use, household income, and an emerging trend in charcoal-burning suicide methods^9^. Other research has reported elevated suicide risk among individuals in long-term care settings^10^ and noted a paradoxical negative association between household debt and suicide in some contexts^11^. However, these investigations largely focus on national-level trends or specific populations, offering limited insight into regional disparities or how suicide risk varies over time and space.

Evidence from other countries demonstrates the importance of subnational analyses in informing public health interventions. In England, for instance, suicide rates vary widely across regions—ranging from 7.9 to 13.8 per 100,000^12^ —and are strongly associated with area-level socioeconomic deprivation^12,13^. Such spatial heterogeneity has also been documented in India, where better-developed and better-educated states often report higher suicide rates, partly due to improved data reporting systems^4^. Research from Japan further also suggests the value of developing suicide surveillance indicators at the local level to assess the effectiveness of community-based prevention strategies and inform regional mental health policy^3^.

In Thailand, data from a 2008 national household survey indicated that 58.5% of individuals experiencing depressive symptoms were also at risk of suicide^14^. Suicide has been linked to a combination of physical and mental illness, relationship breakdowns, and economic stressors^15,16^. The rise in mental health service utilization following the onset of the COVID-19 pandemic highlights an increasing demand for psychological support, likely driven by the pandemic’s wide-reaching impacts on mental well-being^17^. Public health measures—including quarantine, social isolation, school and workplace closures, and economic disruptions—disproportionately affected vulnerable and low-income populations, exacerbating risks of psychological distress, depression, and suicide^8^. While international research has offered important insights into suicide epidemiology, their generalizability to Thailand is limited by differences in sociocultural context, health infrastructure, and data quality.

Existing Thai studies have predominantly focused on national-level trends or specific subpopulations, leaving regional disparities and spatiotemporal patterns largely underexplored. Moreover, few have examined how these patterns relate to contextual risk factors, in part due to limitations in spatial and temporal data granularity. To address this gap, our study aimed to investigate the spatial and temporal patterns of suicide across Thailand’s provinces using a Bayesian spatiotemporal modeling framework. Specifically, we sought to describe long-term trends and geographic variations in suicide rates from 1997 to 2021, identify high-risk areas through cluster detection, and examine associations between suicide rates and selected socioeconomic and demographic factors at the provincial level. We hope these findings can contribute to more locally informed, evidence-based strategies for mental health service planning and suicide prevention in Thailand.

## Methods

### Study design and data sources

This study conducted a retrospective analysis of suicide epidemiology in Thailand using data derived from death certificate records. Suicide cases were classified by province based on the location of death as recorded on the certificates, sourced from the National Suicide Prevention Surveillance Center under the Department of Mental Health, Ministry of Public Health (MOPH), Thailand. The dataset comprised annual provincial-level suicide counts from 1997 to 2021. To support the analysis of potential risk factors, socioeconomic indicators and population data were obtained from the National Statistical Office of Thailand (NSO), both publicly available. To complement the suicide data, geographic coordinates and Thai provincial boundaries data were obtained from the GEO package file in the Global Administrative Region Database (GADM), a comprehensive territorial database that includes sub-national scale data. The data processing and analysis were conducted using R version 4.2.1 and Python version 3.8 programming languages.

### Spatiotemporal multilevel modeling and model selection procedure

#### Spatiotemporal Bayesian model specification

To account for spatial and temporal variation in suicide risk across Thailand, we applied a Bayesian hierarchical Poisson regression model with spatiotemporal random effects which allows for more accurate risk estimation by smoothing unstable standardized mortality ratios (SMRs). Traditional methods often treat geographic units independently, ignoring spatial or temporal correlation and leading to unstable estimates in areas with small populations^18,19^. In contrast, Bayesian modeling allows for smoothing by borrowing strength across neighboring provinces and time periods, improving estimate precision^20^.

Our model included an offset for population size, fixed effects for selected risk factors, and spatially structured and unstructured random effects using the Besag-York-Mollié (BYM) convolution model. Temporal dependencies were accounted for using random walk priors, and various space-time interaction structures were considered to capture potential dependencies across spatial and temporal dimensions. This hierarchical approach facilitates a nuanced analysis of differential suicide risk across provinces and over time. Details on model implementation, prior assumptions, and computational methods are provided in supplementary document S1.

Spatiotemporal cluster detection was performed to identify high-risk areas and time periods for suicide, thereby supporting more targeted public health interventions. Model-based approaches have been shown to outperform traditional methods in detecting spatial and spatiotemporal clusters in public health surveillance^21,22^. In this study, we applied a model-based framework using exceedance probabilities to detect suicide clusters. This approach compares the posterior distribution of estimated suicide counts in each province and year against expected rates. Areas with elevated exceedance probabilities represent significant deviations from expected values and may warrant closer investigation and policy response. Further details on the computational implementation are provided in the supplementary document S1.

#### Model assessment and selection

We conducted an extensive analysis of suicide data in Thailand, applying multiple model specifications to capture spatiotemporal variations and identify associated risk factors. We aimed here to select the most appropriate model that both accurately fits the observed data and offers reliable insights into the incidence and determinants of suicide. The model selection process followed a structured two-step approach^23–25^. In the first step, we identified candidate fixed effects by reviewing a wide range of potential risk factors. These were selected based on their theoretical relevance and empirical support in previous systematic reviews of suicide-related risk factors at both global and national levels^9,11^. We prioritized variables that reflect socioeconomic and demographic conditions, focusing on those consistently reported by the National Statistical Office of Thailand. These included indicators such as household income, debt, poverty levels, and crime rates, which have been shown to influence suicide risk in prior studies^9,11^. To ensure data quality and compatibility with the suicide dataset, we retained only variables with complete or near-complete coverage across all 77 provinces for the entire study period (1997–2021). Specifically, variables with more than 5% missing values were excluded from analysis to reduce potential bias and ensure reliability in spatiotemporal modeling. By applying this threshold, we aimed to balance data completeness with epidemiological relevance. Additional information on the screening criteria and handling of missing data is provided in supplementary document S2.1. This selection process supported the development of a robust fixed-effects structure for the analysis.

Next, in the random-effect selection step, we evaluated multiple combinations of space-time fixed and random effects using various model fit metrics, including the deviance information criteria, bias, root mean squared error (RMSE), and correlation coefficient^26,27^. For specifying the space-time Bayesian models, we considered three types of spatial random effects, three types of temporal effects, and four types of spatiotemporal interaction terms described in Sect. 2.2.1. This process involved evaluating a total of 36 combinations to determine the optimal spatiotemporal mixed structure for suicide modeling. Our objective was to evaluate the most effective spatiotemporal mixed structure for modeling out of those combinations. Throughout the model selection, we aimed to strike a balance between statistical accuracy and epidemiological relevance, ensuring that our findings could be practically applied to develop effective strategies for suicide prevention. Incorporating a Bayesian framework allowed us to consider uncertainty quantification, and we deemed coefficients as significant when their exceedance probability exceeded the predefined level of 0.05. Additional information regarding model comparison and evaluation metrics can be found in supplementary document S2.2.

## Results

### Space-time suicide trends in Thailand

Table 1 presents the annual number of suicide deaths and corresponding crude suicide rates per 100,000 population in Thailand from 1997 to 2021. Over the 25-year period, a total of 106,955 suicide deaths were reported, with an average crude suicide rate of 7.14 per 100,000. A persistent sex disparity is observed: males accounted for 78% of all suicide deaths (*n* = 83,627), with a significantly higher average crude rate of 11.22 per 100,000, compared to 3.16 per 100,000 for females (*n* = 23,328).

**Table 1:** Number of national suicide deaths and crude suicide rates per 100,000 population in Thailand, 1997–2021.

Year	Crude rate			Suicide case		
	Total	Female	Male	Total	Female	Male
1997	7.18	11.09	3.38	4183	971	3212
1998	8.09	12.54	3.63	4964	1121	3843
1999	8.84	13.64	4.09	5290	1210	4080
2000	8.66	13.43	3.96	5189	1151	4038
2001	8.29	12.61	4.04	4803	1137	3666
2002	8.29	12.51	4.13	4905	1190	3715
2003	7.61	11.64	3.64	4486	1060	3426
2004	7.24	11.10	3.45	4296	1043	3253
2005	6.78	10.44	3.19	3941	908	3033
2006	6.24	9.91	2.64	3612	756	2856
2007	6.41	10.02	2.86	3756	812	2944
2008	6.52	10.03	3.07	3778	872	2906
2009	6.42	9.97	2.96	3787	861	2926
2010	6.36	9.97	2.85	3761	848	2913
2011	6.44	9.95	3.02	3873	888	2985
2012	6.87	10.53	3.30	3985	930	3055
2013	6.81	10.83	2.89	3940	850	3090
2014	6.70	10.41	3.09	3950	877	3073
2015	6.99	11.34	2.77	4205	839	3366
2016	6.68	10.69	2.80	4131	848	3283
2017	6.36	10.34	2.50	3934	759	3175
2018	6.73	10.85	2.74	4137	810	3327
2019	7.11	11.85	2.51	4418	799	3619
2020	7.50	12.37	2.80	4821	894	3927
2021	7.50	12.44	2.75	4810	894	3916
**Total**	**178.61**	**280.51**	**79.06**	**106,955**	**23,328**	**83,627**
**Average**	**7.14**	**11.22**	**3.16**	**4278**	**933**	**3345**

The national suicide rate peaked in 1999 at 8.84 per 100,000, largely driven by a male rate of 13.64, while the female rate reached 4.09, the highest recorded for females during the study period. Following this peak, suicide rates declined steadily, reaching a low of 6.24 per 100,000 in 2006. Between 2015 and 2021, there was a modest resurgence, with rates stabilizing around 7.50 in the final two years. Female suicide rates remained relatively stable, fluctuating narrowly between 2.50 and 4.13 per 100,000, whereas male rates demonstrated greater variability and were the primary driver of national trends.

**Fig. 1:**
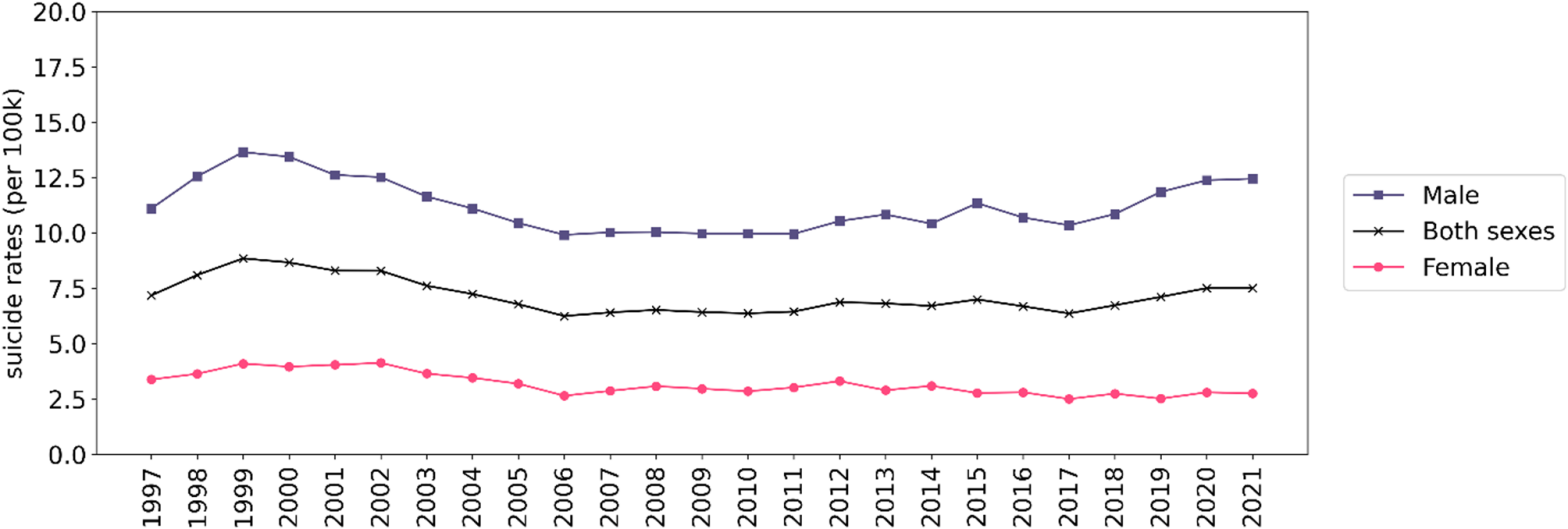
Plot of trends in Thai crude suicide rates by sex duirng 1997–2021 per 100,000 population.

Temporal trends in crude suicide rates by sex are shown in Fig. 1. The national suicide rate peaked in 1999 at 8.84 per 100,000, largely driven by the male suicide rate, which reached 13.64 per 100,000. This peak was followed by a gradual decline over the subsequent decade, reaching a low of 6.24 per 100,000 in 2006—representing a 29.4% decrease. Between 2006 and 2018, rates remained relatively stable. However, a modest but sustained increase was observed from 2018 onward, reaching 7.50 per 100,000 by 2021.

**Fig. 2:**
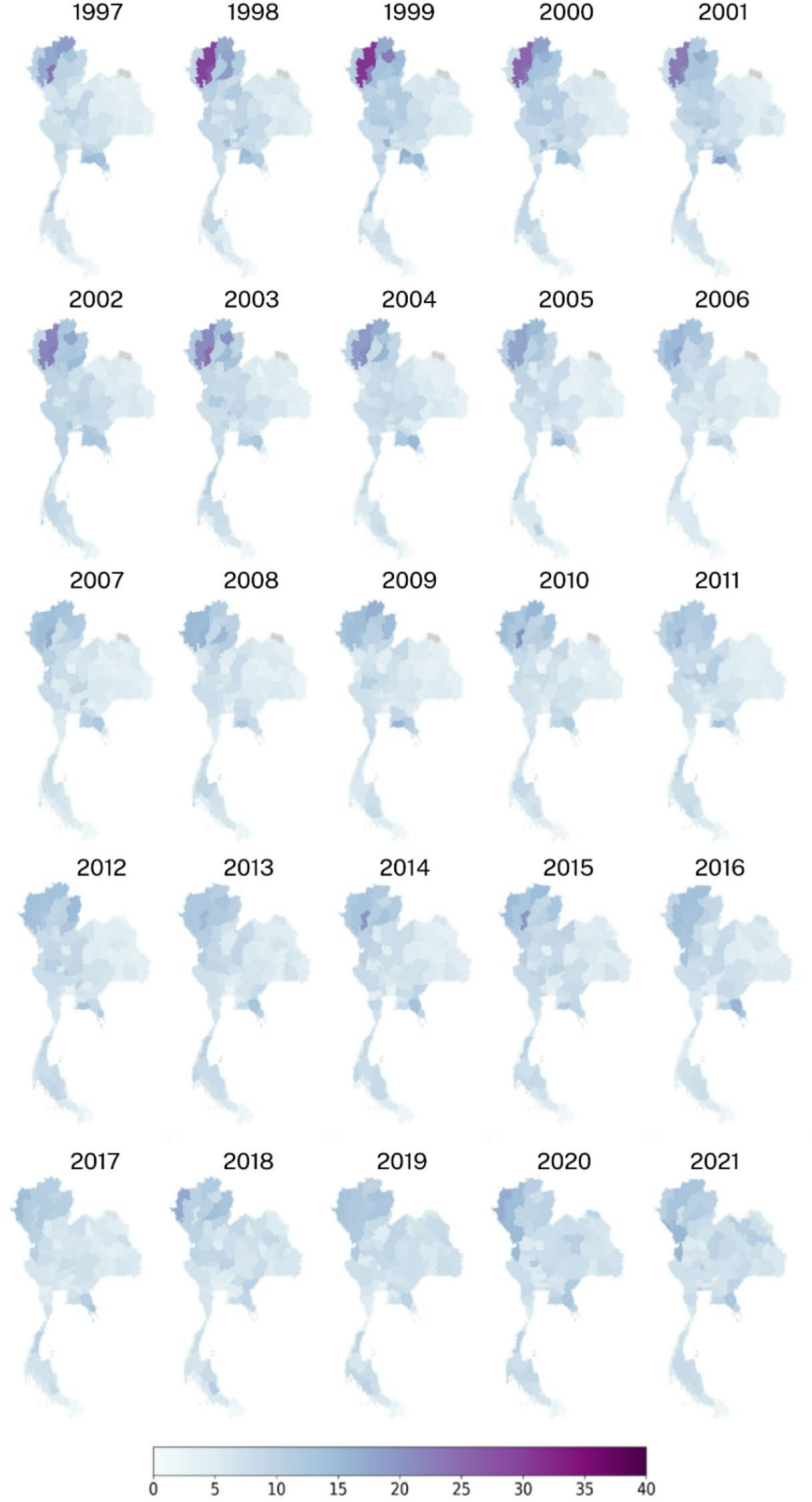
Annual crude suicide rates per 100,000 population by province in Thailand, 1997–2021, generated using RStudio version 2022.07.0 + 548 (available at https://posit.co/products/open-source/rstudio/).

Figure 2 presents annual maps of crude suicide rates per 100,000 population across Thai provinces from 1997 to 2021. In the early years—particularly between 1997 and 2000—elevated rates were concentrated in the northern provinces, with several exceeding 30 per 100,000 and peaking in 1999. From 2001 onward, a general decline in both the magnitude and concentration of high suicide rates is observed. High-rate provinces became less common, and map shading shifted toward lighter tones, reflecting broader reductions nationwide. By the 2010 s, rates appeared more spatially diffuse and overall lower. However, intermittent spikes in certain provinces—seen in 2010, 2014, 2018, and 2020—suggest localized or episodic fluctuations.

**Fig. 3:**
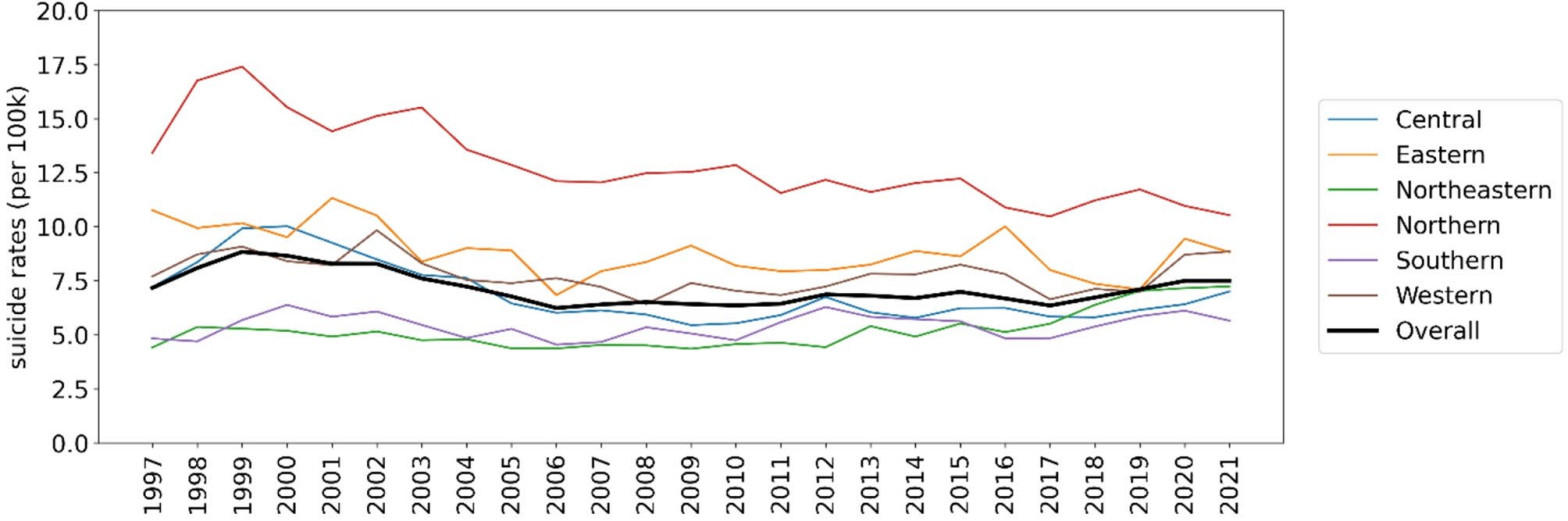
Thai crude suicide rates by region from 1997 to 2021 per 100,000 population.

Figure 3 displays the temporal trends of Thai crude suicide rates by region per 100,000 population. The highest average crude suicide rates were observed in the northern region, starting at 13.42 deaths per 100,000 in 1997 and peaking at 17.41 deaths per 100,000 in 1999, followed by a subsequent decrease of 39.52% to 10.53 deaths per 100,000 in 2021. In comparison, the eastern, western, and central regions exhibited average crude suicide rates close to the overall average, hovering around 9 deaths per 100,000 in 1997 and decreasing to 8 deaths per 100,000 in 2021. The northeastern and southern regions had the lowest average crude suicide rates, but experienced a 39.13% increase from 4.60 deaths per 100,000 in 1997 to 6.40 deaths per 100,000 in 2021.

**Fig. 4:**
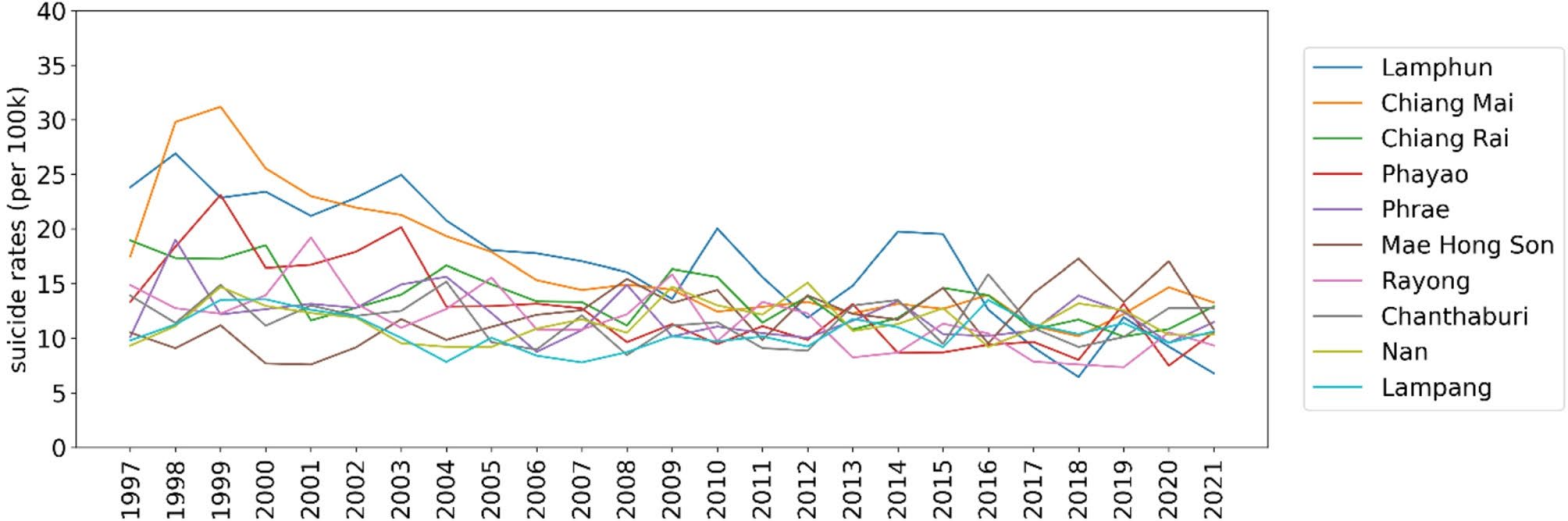
Line chart displaying the top 10 Thai provinces with highest crude suicide rates from 1997 to 2021 per 100,000 population.

Figure 4 presents the top 10 Thai provinces with the highest crude suicide rates from 1997 to 2021 per 100,000 population, with a focus on the provinces in the northern region, namely Lamphun, Chiang Mai, Chiang Rai, Phayao, and Phrae. At the beginning of the study period, Lamphun had the highest crude suicide rates, recorded at 23.80 deaths per 100,000 population, which subsequently decreased over time. Notably, both Chiang Mai and Chiang Rai experienced a significant 28.02% decline in their crude suicide rates, decreasing from approximately 18.20 deaths per 100,000 in 1997 to 13.10 deaths per 100,000. In contrast, Phayao and Phrae provinces displayed relatively stable crude suicide rates, ranging from 10 to 13 deaths per 100,000 throughout the study period.

These findings collectively reinforce the prominence of the highest crude suicide rates in the northern region, consistent with the regional trends observed in Fig. 3. The spatial and temporal distributions of suicide rates depicted in Figs. 2 and 3, and 4 offer valuable insights into the variation of suicide patterns across different regions and time periods in Thailand. The supplementary document S3 provides further descriptive analysis results, including the top 5 provinces for each region.

**Fig. 5:**
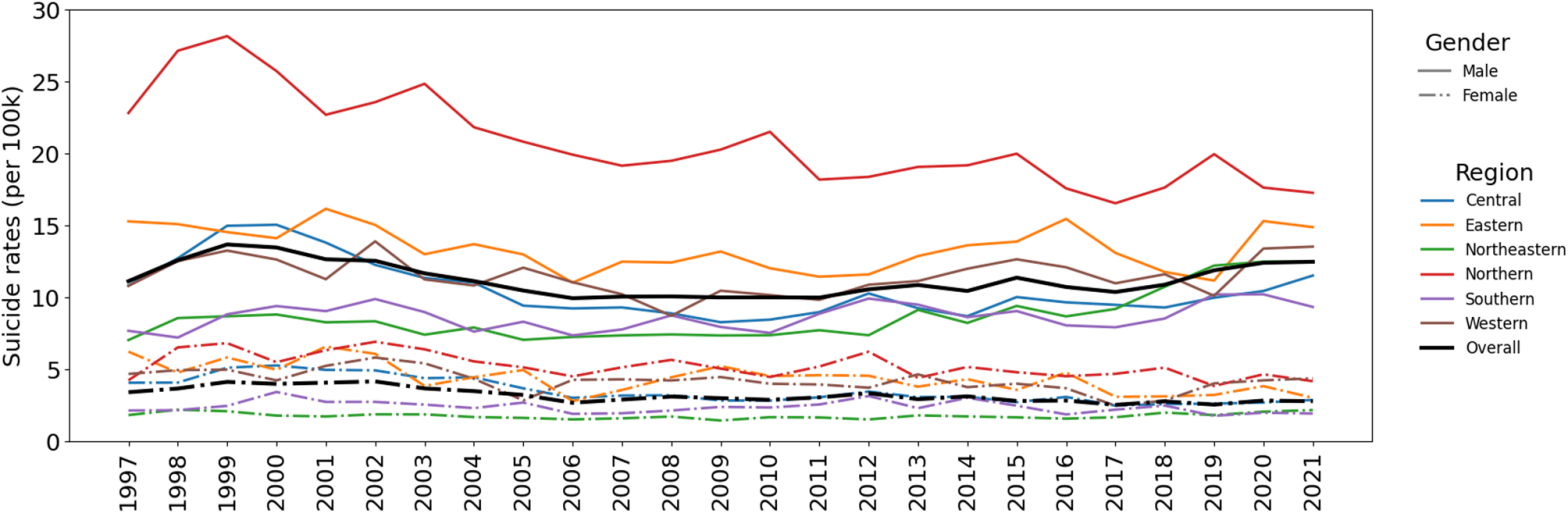
Crude suicide rates in Thailand by region from 1997 to 2021 per 100,000 population. The solid line displays male suicide rates, while the dash-dot line shows female.

In Fig. 5, notable differences were observed between the male and female Thai crude suicide rates from 1997 to 2021 per 100,000 population. Throughout the study period, the overall average crude suicide rates were consistently higher for males compared to females, aligning with global age-standardized suicide rates. The trends in both male and female crude rates mirrored the overall and regional trends. Specifically, the male crude suicide rates increased by 12.17%, rising from 11.09 deaths per 100,000 in 1997 to 12.44 deaths per 100,000 in 2021. Conversely, the female crude suicide rates exhibited a decline of 18.64%, decreasing from 3.38 deaths per 100,000 in 1997 to 2.74 deaths per 100,000 in 2021. These trends are also illustrated in figures S3.2-S3.3 in the supplementary document S3. Moreover, both male and female crude rates showed the highest values in the northern and eastern regions, consistent with the provincial and regional trends highlighted in the study. The northern region in particular had much higher rates in males than the other regions. The sex-specific variations in suicide rates emphasize the need for targeted interventions and prevention strategies that address the underlying risk factors affecting each group. Further exploration of these factors is warranted to develop effective suicide prevention efforts tailored to the specific needs of both male and female populations in Thailand.

### Suicide hotspots analysis and association with risk factors

#### Suicide cluster detection in Thailand

**Fig. 6:**
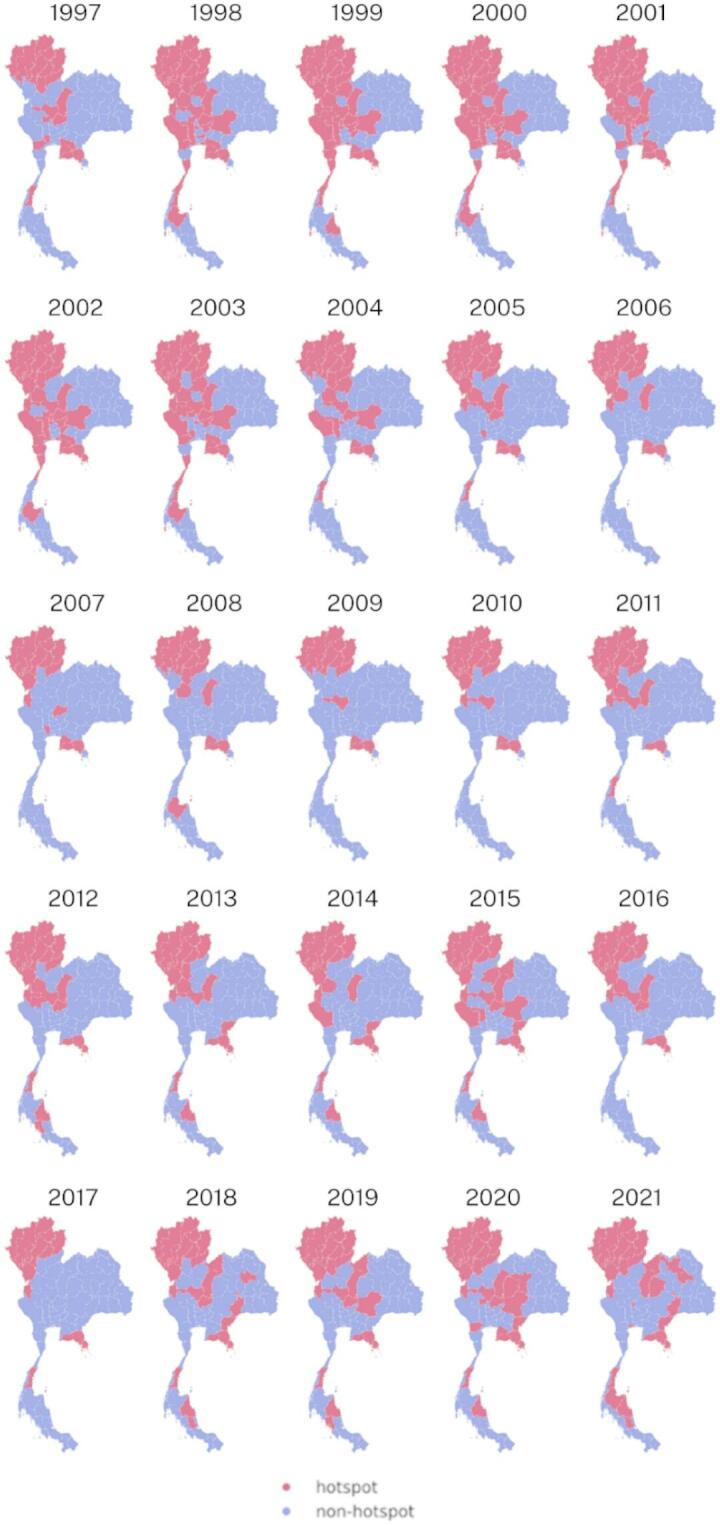
Maps of hotspots of crude suicide rates in Thailand during the years 1997–2021, generated using RStudio version 2022.07.0 + 548 (available at https://posit.co/products/open-source/rstudio/).

The results of the suicide cluster detection analysis offer valuable insights into the spatiotemporal patterns of suicide hotspots across different regions in Thailand. Figure 6 presents the spatial and temporal patterns of suicide hotspots in Thailand. In 1997, the northern region exhibited the highest concentration of suicide rates for both sexes, while the central and eastern regions had relatively low occurrences of suicide hotspots. Between 1999 and 2004, suicide hotspots began to emerge in the western region. Subsequently, the occurrence of suicide hotspots in both the central and western regions started to decline in 2005, and by 2017, suicide hotspots were primarily concentrated in the northern and eastern regions. Notably, since 2018, several provinces in the northeast and south have emerged as new suicide hotspots.

For male suicide hotspots, the results indicated a substantial concentration in the northern region in 1997, with no areas showing high rates of suicide in the northeast or south. The concentration of suicide hotspots in the central region increased from 1998 to 2002 and gradually decreased from 2003 to 2011. Since 2018, new suicide hotspots have emerged in several provinces in the central, northeastern, and southern regions. Regarding female suicide hotspots, between 1997 and 2005, they were concentrated in the north, central, eastern, and western regions. However, suicide hotspots in the central and western regions began to decline in 2006, and by 2017, they had almost entirely disappeared. Additionally, there have been very few suicide hotspots in the northeast and south since 2006 up to the present time.

By identifying these high-risk areas, policymakers and mental health practitioners can strategically allocate resources and implement targeted suicide prevention interventions in areas with the highest concentration of suicides. However, the changing distribution of suicide hotspots over time also highlights the dynamic nature of suicide epidemiology in Thailand, emphasizing the need for adaptive and responsive prevention strategies to address emerging suicide clusters in new regions. The detailed cluster detection plots for each sex in the supplementary document S5 provide further granularity and can inform more tailored suicide prevention efforts for specific populations.

#### Spatiotemporal association with risk factors

In our analysis of the association of risk factors with Thai suicide rates, we explored several socioeconomic risk factors. The first group of risk factors was financial, including monthly household income, expenses, and debt. The second group examined the proportion of poverty in the population, while the third group focused on criminal offenses, encompassing property crimes like thefts and robberies, as well as homicides and other violent crimes such as assaults. To identify the most appropriate model, we compared various combinations of space-time fixed and random effects based on their fit metrics to evaluate the top-performing models for each dataset. The BYM model with a random walk order 1 for the spatial effect and interaction type I was chosen for the combined sex analysis. For males, the BYM model with a random walk order 2 for the spatial effect and interaction type I was employed, and for females, the Besag model with a random walk order 1 for the spatial effect and interaction type I was used. Based on the best models, the analysis revealed significant associations between financial factors and criminal offenses with suicide risk in all three groups of risk factors studied. For each dataset, the best random effects were constructed using financial factors (monthly income per household, monthly expenses per household, and household debt), poverty rate, and criminal offenses factors (theft, robbery, homicide, assault, and violent crimes) to compute the association coefficients or relative risks. The details of the model evaluation for analyzing suicide data and associated risk factors are provided in supplementary document S4.

**Fig. 7:**
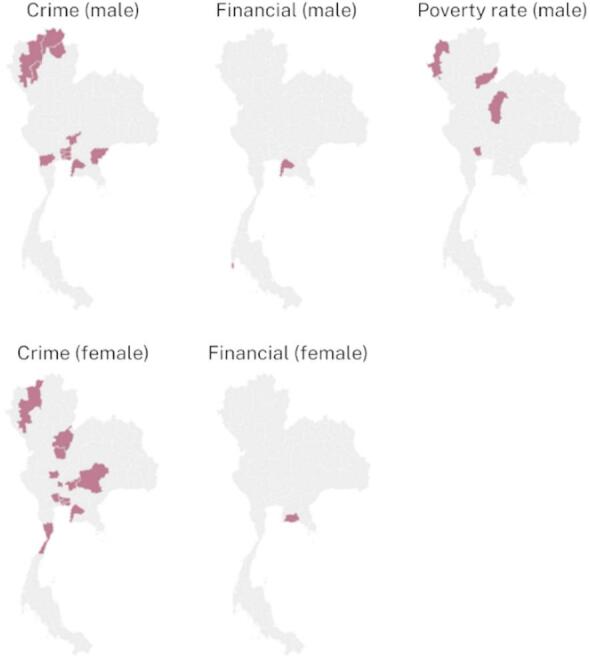
Provincial maps representing the significant socioeconomic factors by sex, with positive associations indicated by red, generated using RStudio version 2022.07.0 + 548 (available at https://posit.co/products/open-source/rstudio/).

As depicted in Fig. 7, the associations between socioeconomic factors and suicide risk displayed spatial variations. Financial factors were significantly associated with suicide risk in provinces, with some provinces showing an increase in suicide risk with increasing financial factors (indicated by red). Notably, the spatial associations varied between males and females. Similarly, the association between poverty and suicide showed spatial variations. However, poverty did not show a spatial association with female suicide data, suggesting the presence of other influential factors in explaining patterns of female suicide rates in those areas. The relationship between criminal offense factors and suicide risk also presented spatial variations. Criminal counts were significantly associated with higher suicide risk in 16 out of 76 provinces for females and in 12 out of 76 provinces for males. Detailed provincial maps highlighting the significant socioeconomic factors for both sexes are provided in the supplementary document S6. These findings offer valuable insights into the spatial dynamics of suicide risk factors in Thailand, informing targeted public health interventions and policies aimed at reducing suicide incidence and promoting mental well-being.

## Discussion

The findings of this study offer important insights into the spatial and temporal distribution of suicide in Thailand between 1997 and 2021. A notable nationwide increase in suicide rates followed the 1997 economic downturn, commonly referred to as the Tom Yum Kung Crisis, underscoring the significant impact of macroeconomic conditions on mental health outcomes^28^. Among all regions, the northern provinces consistently exhibited the highest suicide rates, with male rates substantially exceeding those of females. This sex disparity aligns with prior research in Thailand suggesting that men may be more vulnerable to the psychological strain associated with rapid social and economic transitions^29,30^. Similar sex differences have been reported in countries such as Australia and others globally, where suicide rates are typically higher among males. Contributing factors include mental health conditions, substance use, relationship difficulties, social isolation, and alcohol misuse^31^. Suicide among men is shaped by a complex interplay of individual, societal, and structural influences, underscoring the need for sex-sensitive approaches that foster trust, build protective factors, and incorporate the lived experiences of men within both clinical and community-based settings.

The persistently elevated suicide burden in northern Thailand, consistent with previous research^32,33^, appears to be shaped by a complex interplay of sociocultural, economic, and psychosocial determinants. A regional study reported that hanging was the most common method of suicide, followed by ingestion of agricultural chemicals, with the highest incidence concentrated in the upper northern provinces^33^—findings consistent with our spatial analysis. Qualitative research in these areas has further identified key risk factors among individuals with a history of suicide attempts, including recurrent family conflict, unstable interpersonal relationships, inadequate coping mechanisms, and the use of alcohol or other substances to alleviate stress^30^. In some provinces, these individual-level stressors are compounded by broader social vulnerabilities. For example, a study in Chiang Mai identified financial hardship and living with chronic health conditions, such as HIV, as contextual factors associated with increased suicide risk^33^. Although northern Thai communities are often characterized by strong social cohesion and cultural values such as kindness and hospitality, these findings suggest that such community-level narratives may mask deeper, structural challenges affecting mental well-being.

Financial factors were found to be linked to a rise in suicide risk in various provinces, particularly in the north and central regions. However, in some areas, the number of suicides decreased, possibly due to increased income being used to pay off rising debt, preventing an increase in suicides. In other provinces, an increase in financial issues was associated with an increase in suicides, possibly due to financial problems being a significant source of stress and anxiety, leading to depression, hopelessness, and despair, which are known risk factors for suicide. Moreover, financial difficulties may have limited access to resources such as mental health services or medication, exacerbating mental health problems and increasing the risk of suicide. Studies on the relationship between risk factors and suicide in Thailand are not abundant, but evidence suggests that financial issues are connected to suicide. During the economic crisis in Thailand between 1997 and 2000, a higher rate of unemployment was linked to an increase in suicidal thoughts, particularly in the north and central regions^28^. Research in other countries, like South Korea^34^ and the United States^35^, has also shown significant associations between unemployment, low income, and debt with suicide risk.

The finding that criminal offenses are significantly associated with elevated suicide risk in several provinces for both males and females—though with differing geographic patterns—raises important questions about the mechanisms underlying these associations. While such relationships were not observed in the Thai context at the population level, prior studies have documented co-occurrence of sexual assault, physical violence, and suicidal behavior within intimate partner relationships in specific subpopulations^36^. Observed sex-specific associations in other contexts indicate that males and females may experience or respond to violence and crime in distinct ways. For example, research has demonstrated that women who experience intimate partner violence are at heightened risk of depression and suicidal behavior^37^.

However, broader regional evidence remains limited. A study conducted in Southeast Asia reported that fewer than 10% of suicide cases were related to homicide, and did not examine links with other forms of criminal offenses^38^. In contrast, research from Australia has shown that suicidal ideation and behavior frequently co-occur with a range of health and social risk behaviors, underscoring the need for integrated public health interventions that bridge clinical care and community-level suicide prevention strategies^39^. It is important to note, however, that the presence of criminal activity does not necessarily imply a causal relationship with suicide risk. In some cases, suicidal ideation may precede or co-occur with engagement in criminal behavior, reflecting broader psychosocial distress or diminished regard for personal safety. These findings point to a complex, bidirectional relationship that warrants further investigation.

The observed associations between suicide risk and financial factors, as well as crime-related variables, are consistent with previous research elsewhere indicating that economic strain and social instability are important contributors to suicidal behavior^40^. However, the current study did not explore potential nonlinear effects or interactions between these risk factors, which may have important implications for understanding suicide dynamics. For example, financial hardship may not only directly contribute to psychological distress, but its impact may be amplified in contexts where access to mental health services is limited. Studies have shown that individuals in economically disadvantaged areas often face barriers to mental healthcare, compounding the risk of suicide^13^. Future research could benefit from employing more complex modeling approaches—such as interaction terms or nonlinear functional forms—to investigate how economic stressors may interact with health system accessibility and social safety nets to influence suicide risk. In addition, while the spatiotemporal modeling captured important geographical and temporal trends, there are limitations in terms of the variables included. Factors such as internal migration, demographic shifts, urbanization, and evolving mental health policies were not explicitly modeled but may have contributed to observed patterns. For example, the expansion of the universal health coverage scheme in Thailand in the early 2000 s and subsequent mental health reforms may have influenced service accessibility over time, potentially altering suicide risk across regions^41,42^.

Although the analysis incorporated key socioeconomic indicators, other important confounders and determinants of suicide—such as individual mental health history, access to mental health services, and the strength of social support networks— could not be assessed due to the lack of consistently available data at the provincial level. Including these variables in future research could enhance understanding of the complex interplay of factors contributing to suicide risk. In addition, the analysis was based on data collected through 2021. While this allows for an assessment of long-term trends, it may not fully capture more recent developments, particularly the evolving impact of the COVID-19 pandemic on suicide and mental health. Studies from other countries have documented rising mental health challenges, financial strain, and increased interpersonal conflicts in the post-pandemic period, all of which may contribute to suicide risk^43,44^. In Thailand, a spatiotemporal analysis has reported increases in mental health service utilization, particularly for anxiety, schizophrenia, and depression, following the onset of the pandemic^17^. These findings suggest that shifts in socioeconomic and behavioral patterns during and after the COVID-19 crisis may have introduced new vulnerabilities and altered existing suicide trends. Given these considerations, future research could extend this work by integrating more recent data and incorporating a broader range of risk factors. However, the effect of COVID-19 should be carefully incorporated and carefully interpretation.

In this study, a linear relationship between suicide and potential risk factors was assumed, and potential nonlinear effects or interactions between variables were not explicitly modeled. This may limit the depth of interpretation, as the influence of financial stress, for example, could be intensified in areas with limited access to mental health services or social support. While our model incorporated flexible space–time random effects and interaction terms to capture underlying spatiotemporal variation^23^, it may not fully account for complex interdependencies among risk factors. The precise nature of these relationships also remains underexplored. Therefore, future studies would benefit from employing modeling frameworks that allow for nonlinear associations and interaction effects—particularly to assess how economic factors interact with other determinants, such as healthcare access or social capital, when such data are available.

This study aimed to provide a broad, national-level picture of the spatiotemporal epidemiology of suicide using the most comprehensive data source currently available from the national suicide surveillance system. Similar to other published suicide studies in Thailand^7,8^, we relied on mortality data obtained from death certificates reported through the national suicide prevention surveillance center, managed by the Department of Mental Health. We acknowledge, however, that the quality of suicide mortality data remains a significant limitation. The World Health Organization (WHO) has rated Thailand’s suicide data quality as low reflecting concerns about underreporting and misclassification^45^. Suicide is a highly stigmatized cause of death globally, and the extent of underestimation varies significantly across countries, however this issue is common, especially in low- and middle-income countries^46,47^. In Thailand, a study using verbal autopsy data from 2005 estimated that approximately 43% of suicide deaths were underreported in the national vital registration system, with 11.4% misclassified as ill-defined and 31.7% as other external causes^48^. Variability in reporting practices across provinces and over time—due to differences in administrative capacity, health infrastructure, and cultural attitudes toward suicide—may have introduced bias into the observed trends and spatial patterns. Nonetheless, the spatial distribution of suicides in the 2005 study in Thailand showed patterns similar with our findings, particularly the concentration of higher suicide rates in the northern provinces. This suggests that while the absolute numbers may be underestimated, the relative geographic distribution may still reflect genuine disparities.

Possible sources of underreporting include misclassification of cause of death, particularly for cases occurring outside healthcare settings where reporting often relies on non-medical personnel. Even within hospitals, the use of nonspecific diagnostic codes can contribute to misclassification^49,50^. Given these limitations, the results of this study should be interpreted with appropriate caution. Nevertheless, we believe that the patterns identified—particularly long-term spatial and temporal trends and their associations with key socioeconomic factors—offer important insights. Future research however could strengthen these findings by triangulating multiple data sources to improve suicide monitoring and support more accurate, evidence-informed public health interventions in Thailand.

### Policy implications

This study underscores notable spatial and temporal disparities in suicide rates across Thailand and emphasizes the urgent need for targeted, evidence-based prevention strategies. The consistently elevated suicide burden in the northern provinces highlights the importance of regionally tailored and sex-responsive interventions. Strengthening the integration of mental health services into primary care settings could improve access to care, particularly in rural and underserved regions where mental health infrastructure remains limited^51,52^. In addition, public education also plays a vital role in reducing stigma and encouraging help-seeking behaviors, both of which are essential to suicide prevention^53^. Additionally, media reporting practices significantly influence public understanding and responses to suicide. Irresponsible reporting has been shown to contribute to suicide contagion, making it crucial to train media professionals in safe and ethical reporting practices^54^.

Reliable and timely data are also essential for effective suicide prevention. Strengthening national suicide surveillance systems to capture disaggregated and up-to-date information can better guide interventions and support efficient resource allocation^17,55^. Lastly, addressing the structural drivers of suicide—such as poverty, unemployment, and substance use—requires multisectoral collaboration across health, education, justice, and social welfare sectors^56,57^. By integrating these strategies into a cohesive national framework, Thailand can develop contextually informed and sustainable approaches to reduce suicide and promote mental health equity.

## Conclusion

Given the scarcity of research on suicide in Thailand, this analysis provides new information, unraveling its spatiotemporal complexities and shedding light on potential risk factors. The profound regional disparities and sex-specific trends unveiled through this analysis offer a granular comprehension of the intricate landscape of suicide rates. The importance of high-risk clusters emphasizes the critical need for targeted interventions that consider both geographical and temporal dynamics. The nuanced insights into sex-specific patterns spotlight the necessity of dismantling traditional sex norms and fostering mental health support mechanisms that cater to the distinct needs of various populations.

This work holds significance for informing mental health policies in Thailand. By delving into the intricate relationship between suicide determinants and their spatiotemporal context, this study can provide policymakers with a robust evidence base for formulating effective interventions. Utilizing these insights, policies can be tailored to address region-specific vulnerabilities and temporal variations, ultimately contributing to the reduction of suicide rates. The spatiotemporal lens through which this research examines suicide trends in Thailand brings into focus the localized and evolving nature of this complex issue. By incorporating these findings into mental health policies, Thailand can take strides toward cultivating a more resilient and supportive society. In addition, this contribution can be integral to shaping policies that not only curb suicide rates but also enhance the overall mental health landscape of the nation.

## Supplementary Information

Below is the link to the electronic supplementary material.

Supplementary Material 1

## Data Availability

The publicly available datasets used in this study were obtained from the National Suicide Prevention Surveillance Center, managed by the Department of Mental Health, Ministry of Public Health, Thailand (https://suicide.dmh.go.th), and from the National Statistical Office of Thailand (https://www.nso.go.th/nsoweb/index).
