# Supplementary material for: Spatiotemporal epidemiology and hierarchical analysis of suicide mortality and associated risk factors in Thailand using national surveillance data during 1997–2021
Source: Sci Rep. 2025 Oct 9;15:35220. doi: 10.1038/s41598-025-20481-0 (PMC12511324; doi:10.1038/s41598-025-20481-0)
Supplement: Supplementary file 1 — Supplementary Material 1 [file 41598_2025_20481_MOESM1_ESM.docx]

**Supplementary document: Spatiotemporal epidemiology and hierarchical analysis of suicide mortality and associated risk factors in Thailand using national surveillance data during 1997-2021**

**Supplementary document S1: Spatiotemporal hierarchical modeling and hotspot analysis**

To analyze Thai suicide data, suicide death counts ($y_{it})$ for location *i* and time *t* were modeled using a Poisson distribution as $y_{it}\sim Poisson\left( \rho_{it} \right),$ where $\log\left( \rho_{it} \right)=\log\left( E_{it} \right)+\alpha+\Sigma_{m}^{M} \beta_{mit} x_{mit}+\eta_{it}$. Here, $E_{it}$ is the offset term, $x_{mit}$​ represents the risk factor of interest, and $\beta_{mit}$ is the corresponding coefficient of risk factor *m*. The interpretation of the model conditioned on random effect terms and other fixed effects indicated that the overall geometric mean of the age-standardized suicide mortality rate for risk factor *m* as expected to change by $100\left( exp\left( \beta_{mit} \right)-1 \right)$ percent as risk factor $x_{mit}$ increases one unit. The space-time random effects $\left( \eta_{it} \right)$ were incorporated to account for variation due to location and time, where the spatial random effect​ comprised both spatial and non-spatial structures. The unstructured spatial effect $\left( v_{i} \right)$ was modeled with a zero-mean normal prior distribution, and the spatially structured effect $\left( u_{i} \right)$ was specified as the intrinsic conditional autoregressive (ICAR) model developed by Besag et al [1] as $u_{i}|u_{-i} \sim Normal\left( \mu_{i}+\frac{1}{N_{i}}\Sigma_{j=1}^{n}a_{ij}\left( u_{j}-\mu_{j} \right), \sigma_{j}^{2} \right)$. The convolution model (BYM) was specified as combination of unstructured spatial and spatially structured effects.

# Temporal effects included both unstructured effect $\left( \boldsymbol{\phi}_{\boldsymbol{t}} \right)$ and structured effect $\left( \boldsymbol{\gamma}_{\boldsymbol{t}} \right)$ with options for random walk priors of order 1 or 2. This is the temporal terms can be written as $\boldsymbol{\phi}_{\boldsymbol{t}}\boldsymbol{\sim iidNormal}\left( \boldsymbol{0,}\boldsymbol{\sigma}^{\boldsymbol{2}} \right)$, $\boldsymbol{\gamma}_{\mathbf{t}}\boldsymbol{|}\boldsymbol{\gamma}_{\mathbf{t}\boldsymbol{-1}} \boldsymbol{\sim} \mathbf{Normal}\boldsymbol{(}\boldsymbol{\gamma}_{\mathbf{t}\boldsymbol{-1}}\boldsymbol{,} \boldsymbol{\sigma}^{\boldsymbol{2}}\boldsymbol{)}$, and $\boldsymbol{\gamma}_{\boldsymbol{t}}\boldsymbol{|}\boldsymbol{\gamma}_{\boldsymbol{t-1}}\boldsymbol{,}\boldsymbol{\gamma}_{\boldsymbol{t-2}}\boldsymbol{\sim Normal(2}\boldsymbol{\gamma}_{\boldsymbol{t-1}}\boldsymbol{+}\boldsymbol{\gamma}_{\boldsymbol{t-2}}\boldsymbol{,}\boldsymbol{\sigma}^{\boldsymbol{2}}\boldsymbol{)}$. The prior distribution of space-time interaction $\boldsymbol{(}\boldsymbol{\delta}_{\boldsymbol{it}}\boldsymbol{)}$ depended on the spatial and temporal main effects which are assumed to interact. Four types of interactions were incorporated into the Bayesian modeling framework to capture inseparable space-time variation in disease risk, as recommended by [2]. These interactions allowed for an examination of how spatial and temporal factors interacted in influencing the observed data.

A number of diagnostic tools are available to evaluate the local anomalies. However, it is a natural idea to consider a hotspot as any isolated locations or geographically-bounded regions that display an excess of disease risk or incidence in a particular time. The excess of suicide risk can be examined by comparison with the expected rate. There are a number of ways to adjust for the baseline (see examples [3-5]), however a common practice for health mapping [6] is to calculate the expected rate as $E_{it}=\frac{\sum_{i} \sum_{t} y_{it}}{\sum_{i} \sum_{t} pop_{it}}pop_{it}$, where$y_{it}$and$pop_{it}$are the incidence and population at risk for each location and time. We wanted to perform the analysis to find persistent hotspots over the study period, Then the expected rate used in the analysis was computed as $E_{i}=\frac{\sum_{i} \sum_{t} y_{it}}{\sum_{i} \sum_{t} pop_{it}}\overline{pop}_{i}$where $\overline{pop}$is the population at risk in each province averaged over the study period.

Previous studies have demonstrated the superior performance of model-based methods for spatial and spatiotemporal hotspot detection in public health surveillance [7, 8]. Accordingly, this study employed a Bayesian framework for space-time anomaly detection. Specifically, we calculated the exceedance probability, defined as $P(\rho_{it}/E_{it}>a)$> 1-$\alpha$, exceedance probability, from the number of estimates in the posterior sample which exceed a threshold. Usually the limit is assumed to be *a*=1 which means we apply the level of the expected rate as the baseline. In this study, maps of the resulting probability surface were investigated for high-risk areas, at the level of $\alpha$ = 0.05. A high exceedance probability indicates that the risk at that location and time period is higher than expected and could potentially lead to the detection of suicide clusters.

In the Bayesian perspective for calculating suicide incidence in each province and time period, the estimates can be treated as missing data. An effective method for handling this missing information is by utilizing the posterior predictive distribution, providing both point estimates and uncertainty bands. The formulation of the posterior predictive distribution of suicides is given by equation (1), where full data and parameter space represent the data and parameter space used in fitting the model

$p(y_{it}|\tilde{y})=\int_{\Theta} p(y_{it}|\Theta,\tilde{y})p(\Theta|\tilde{y})d\Theta$ (1)

where$\tilde{y}$ and $\Theta$denotes the full data and parameter space used to fit the model.

Estimates from the predictive distribution are typically derived from well-converged posterior samplers using sampling-based algorithms like Markov chain Monte Carlo (McMC). However, due to the numerous linear predictor specifications we aim to compare and the potential rapid expansion of the parameter space in a multi-dimensional model setup, demanding significant computational resources, a more efficient strategy is employed. In this context, the integrated nested Laplace approximation (INLA) [9] offers an effective means of inferring parameters. With relatively fast numerical routines and compatibility of our proposed model with the INLA format, the model selection process was conducted within the framework of the Laplace approximation using the R-INLA package accessible at www.r-inla.org. It's important to note that the prior distribution for the non-spatial coefficient of the fixed time effect was assumed to be a zero-mean Gaussian with a precision of 10^-6^ Simultaneously, all precision parameters for random effects were assumed, on the log scale, to follow a log-Gamma distribution (1, 10^-5^) as the suggested default for INLA.

**Supplementary document S2: Risk factor selection**

**S2.1 Data processing**

The data preparation for Thai suicide data involved two main steps. In the first part, we calculated the crude suicide rate data by multiplying the number of suicides by 100,000, using population proportions as weights. This data spanned 25 years from 1997 to 2021. The challenge was converting the data from PDF to CSV format, necessitating the download of 25 PDF files from the Center for Suicide Prevention website and their conversion to Excel using the Excel Power Query tool. The Excel files were then processed in Python, where we cleaned the data using regular expressions and transformed it into numeric data type. The final version of the data included 26 columns, representing province names, years, and suicide rates for both genders, with separate CSV files for female, male, and both genders. The second step involved preprocessing potential risk factor data from Thailand. We selected seven types of potential risk factors from the National Statistics Office of Thailand, covering Thai-specific factors. To handle missing values in discrete and continuous datasets, we employed data interpolation using Bayesian regression.

In this study, the selection of risk factors was guided by both data availability and their relevance to previously identified determinants of suicide. We prioritized variables that were publicly accessible and consistently reported by the National Statistical Office and other official Thai government sources from 1997 to 2021 to ensure both temporal continuity and geographic completeness. Socioeconomic indicators—such as household income, household debt, and crime statistics—were selected based on their frequent identification as area-level correlates of suicide in prior systematic reviews and relevant studies, particularly within the Thai context [10-13].

To construct a coherent and analyzable dataset, candidate risk factors were screened based on two criteria: alignment with established indicators from the literature and the availability of data for all 77 provinces. In determining an acceptable threshold for missing data, we adopted a conservative approach informed by existing methodological guidance. Prior research suggests that when less than 5% of data are missing, the benefits of multiple imputation are minimal [14]. Conversely, missingness exceeding 10% may introduce bias, and when more than 40% of data are missing for key variables, the results should be interpreted as exploratory or hypothesis-generating only [15, 16]. Based on these recommendations, we retained only those variables with less than 5% missing data for inclusion in the analysis. This criterion ensured that the suicide and risk factor datasets were aligned both geographically and temporally—an essential requirement for robust spatiotemporal modeling. The variable selection and data preparation workflow are depicted in Figure S2.1.

The selected risk factors were grouped into three main categories. The first group included financial indicators—monthly household income, household expenditure, and household debt. The second group represented the proportion of the population living below the poverty line. The third group comprised criminal offense indicators, which included property crimes such as theft and robbery, as well as violent crimes including assault and homicide. These categories were chosen to capture multiple dimensions of social and economic stressors that may contribute to suicide risk across Thai provinces.


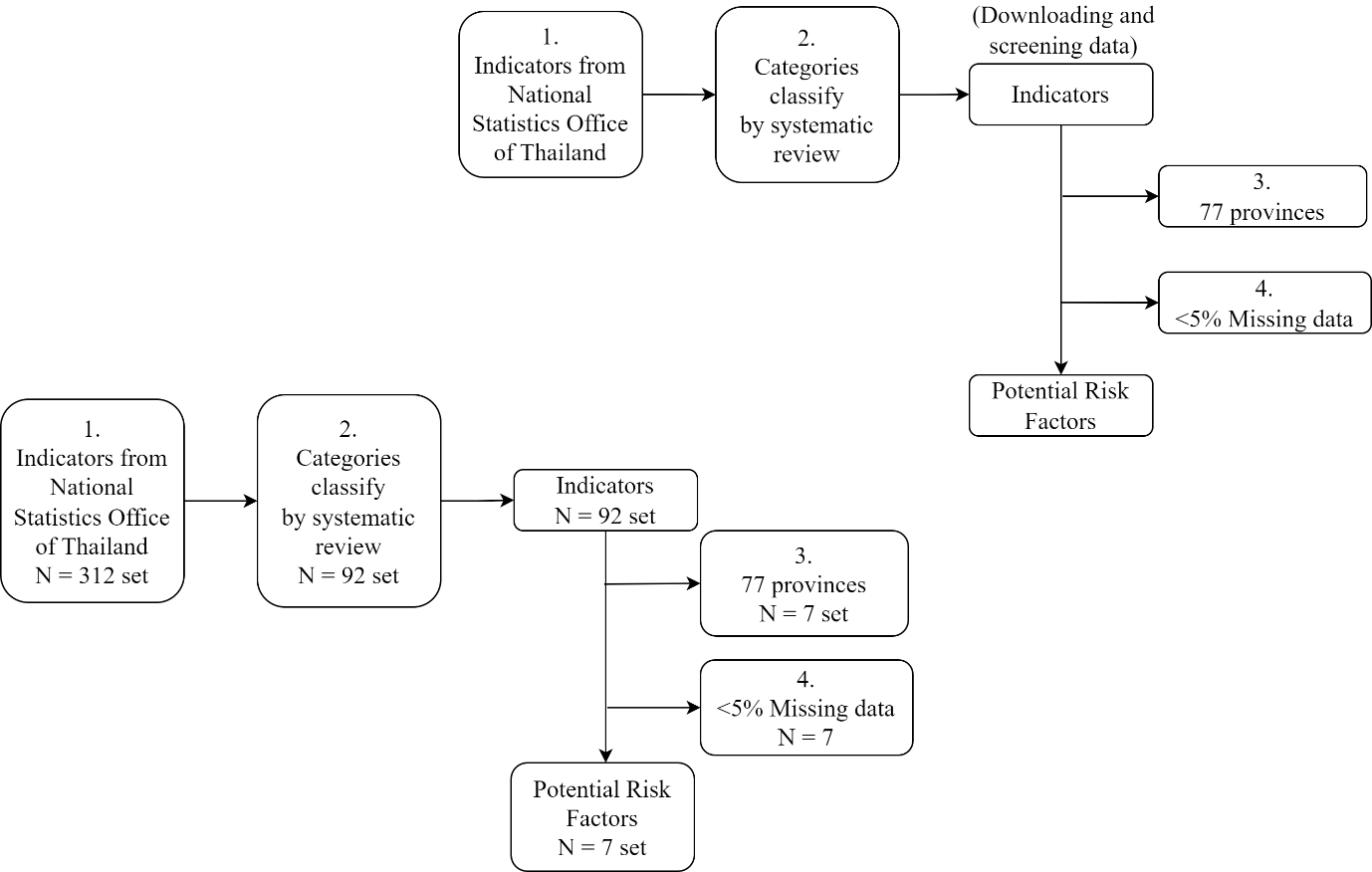


**Figure S2.1** Criteria for the selection of potential Thai risk factors.

**S2.2 Model evaluation metrics**

Various methods were employed to assess the models' performance and determine the adequacy of fitting the data in space and time. Two common goodness-of-fit measures, the deviance information criterion (DIC) [17] and the widely applicable or Watanabe-Akaike information criterion (WAIC) [18], were used based on information criteria. DIC, a Bayesian extension of the Akaike information criterion (AIC) in the Frequentist framework, has been widely employed to assess overall model fit. WAIC, on the other hand, can be viewed as an improvement on DIC, as it fully utilizes the posterior distribution and is robust to different parametrizations, making it valid even for singular models [19].

Additionally, bias, defined as the average difference between the observed incidence and its estimated counterpart across locations and time periods, was computed. A bias close to zero is preferred, indicating a good fit of the model. Estimation uncertainty was also investigated using the root mean squared error (RMSE), which represents the squared root of the average squared deviation between the observed and estimated values across spatial units and study time periods. Lastly, Spearman’s correlation coefficient was calculated to assess the correlation between the observed and estimated health outcomes. These comprehensive measures collectively provided insights into the accuracy and reliability of the models' predictions and their ability to capture the underlying patterns in the data.

**Supplementary document S3: Plots and maps of suicide crude rates by area and gender**

Central


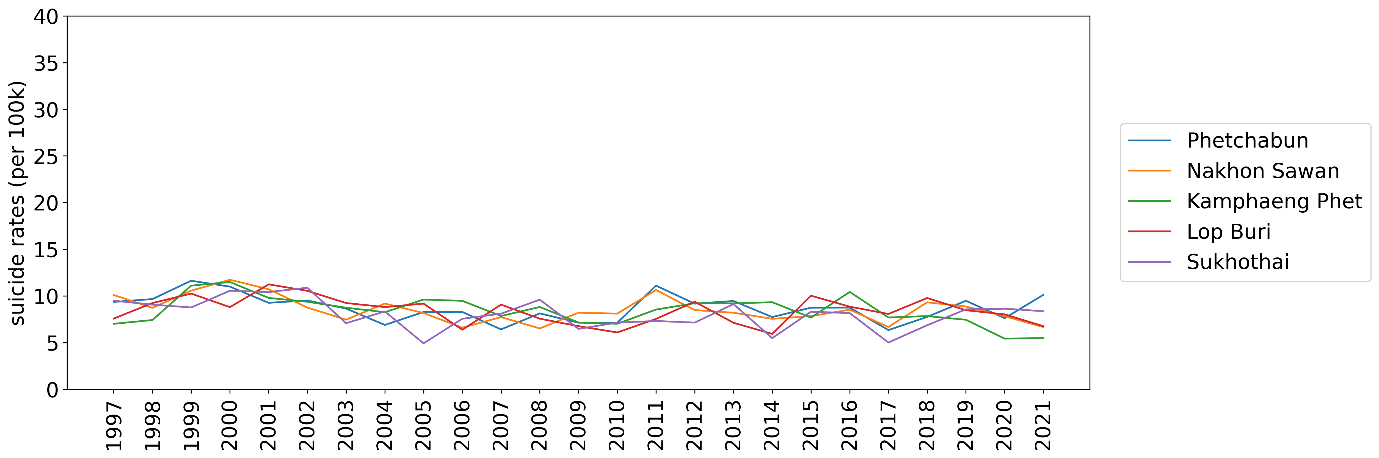


Eastern


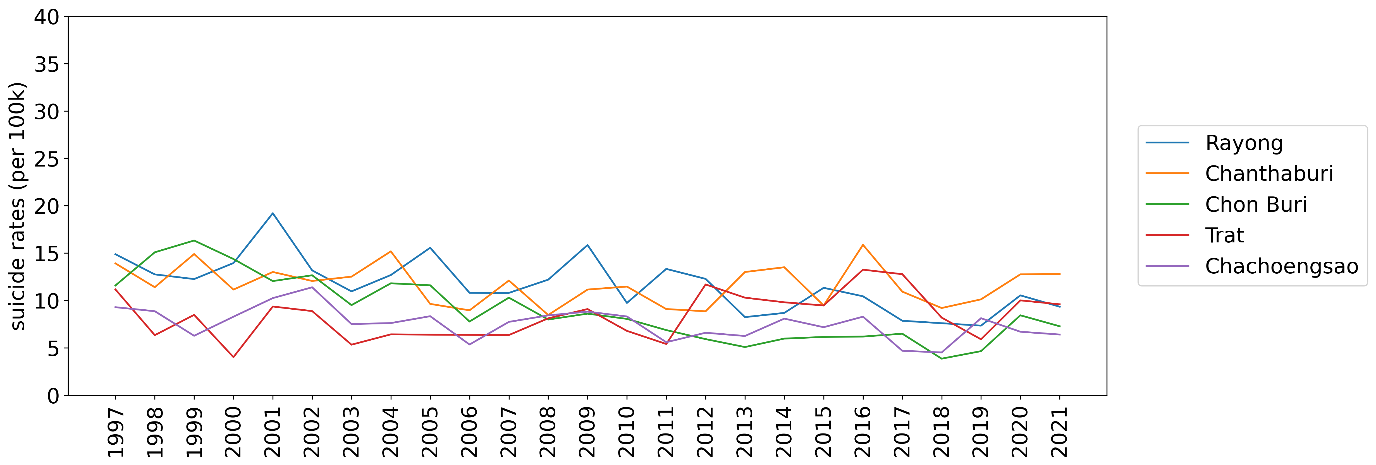


Northeastern


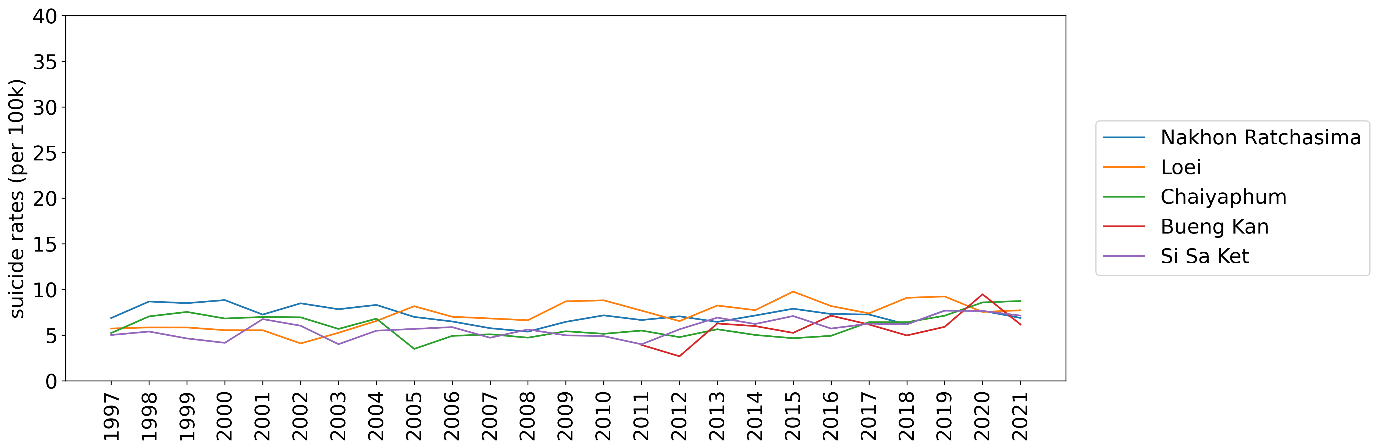


Northern


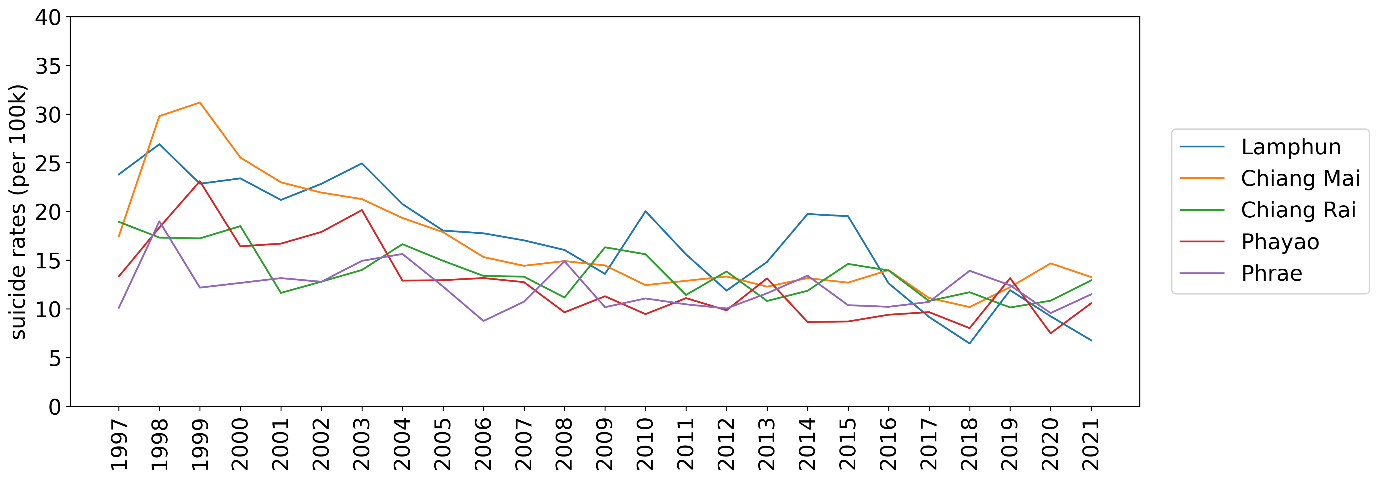
Southern


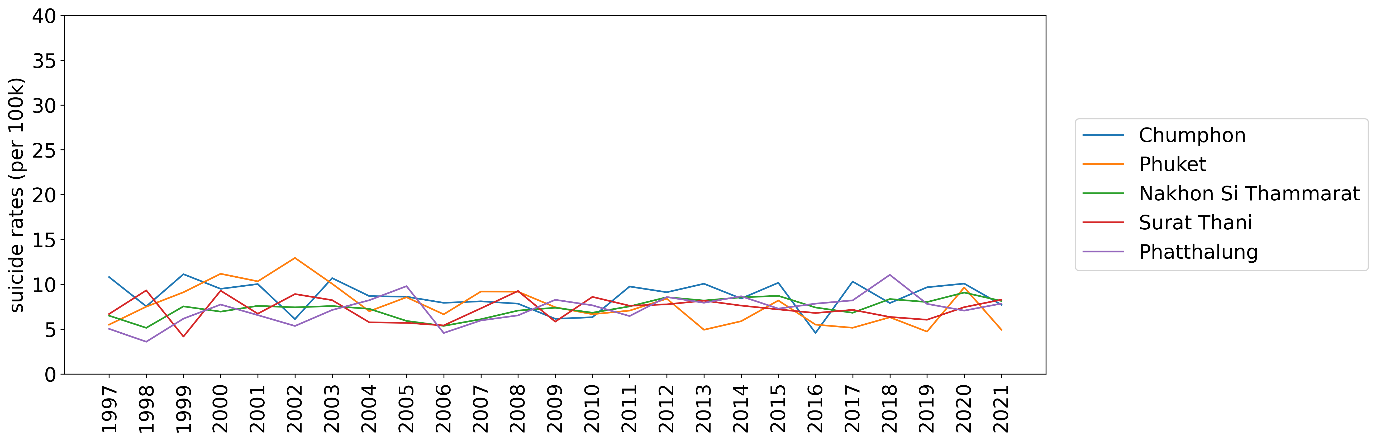


Western


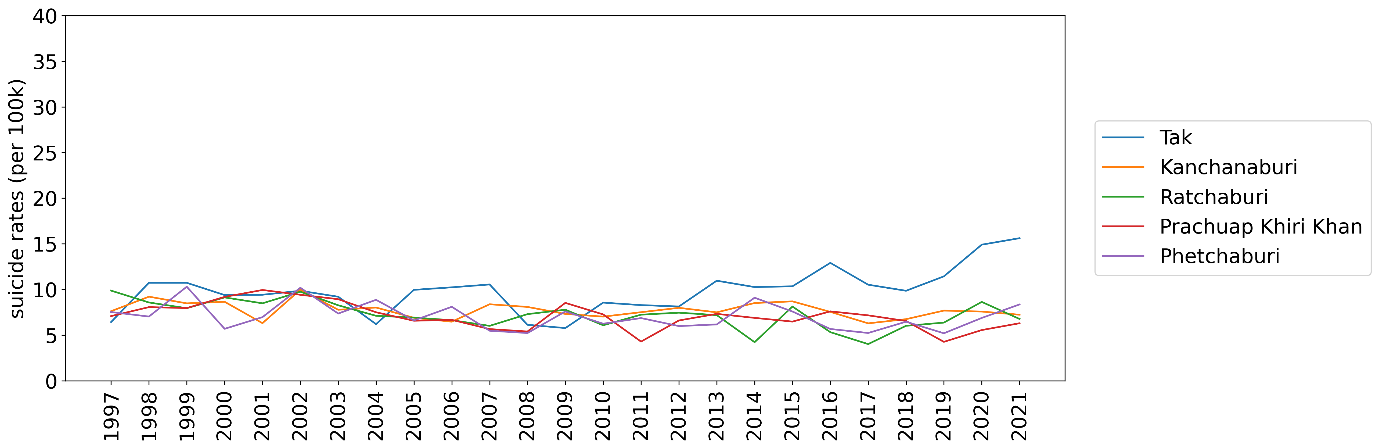


**Figure S3.1** Plots depicting the top 5 provinces in each Thai region, showing crude suicide rates from 1997 to 2021 per 100,000 population.


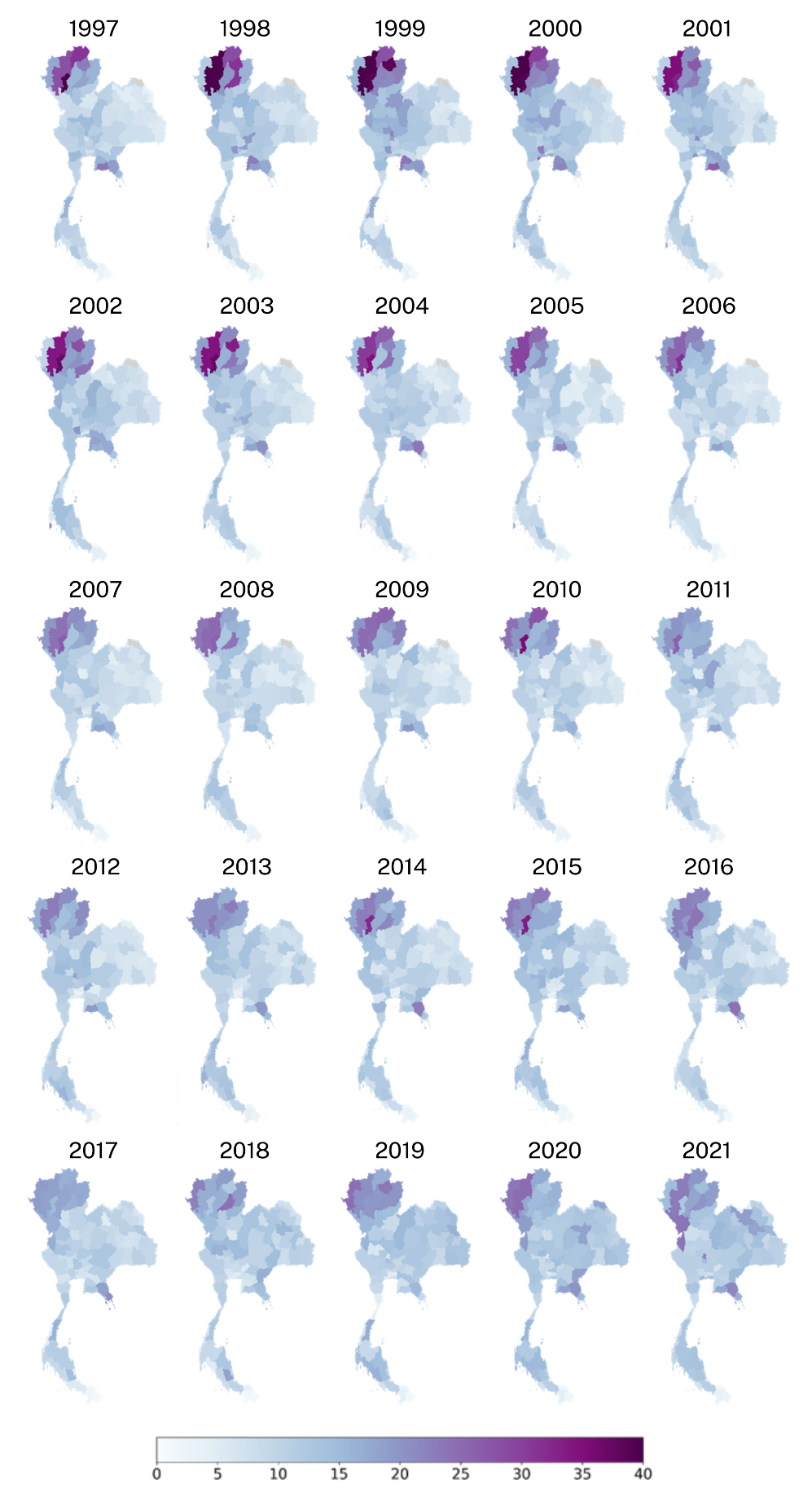


**Figure S3.2** Provincial maps illustrating Thai male crude suicide rates from 2000 to 2019 per 100,000 population, generated using RStudio version 2022.07.0+548 (available at https://posit.co/products/open-source/rstudio/).


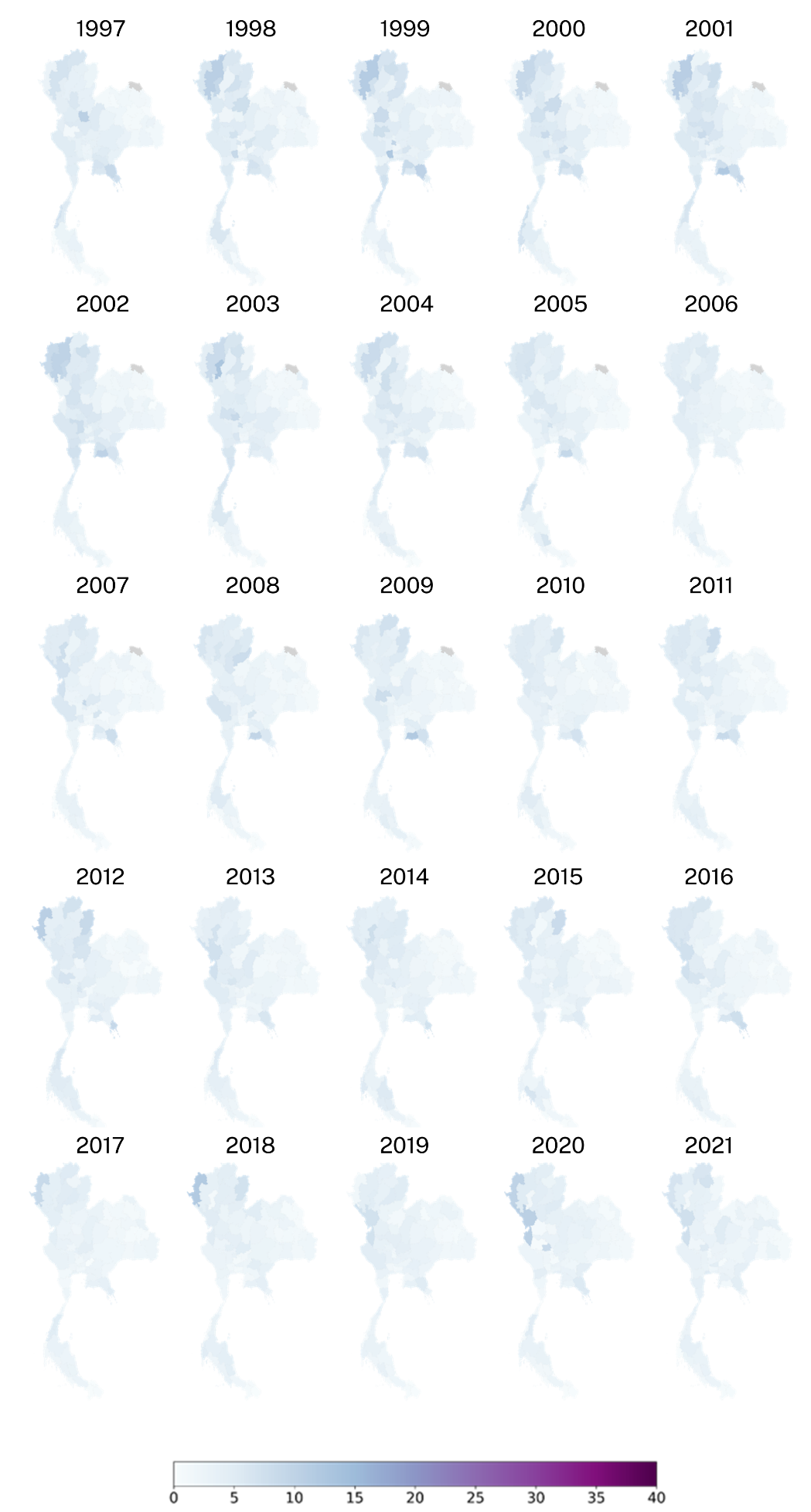


**Figure S3.3** Provincial maps illustrating Thai female crude suicide rates from 2000 to 2019 per 100,000 population, generated using RStudio version 2022.07.0+548 (available at https://posit.co/products/open-source/rstudio/)..

**Supplementary document S4: Results of model evaluation for analysis of suicide data and risk factors, the column header abbreviations and definition list, and the included risk factors list.**

| **model_name** | **bias** | **mse** | **dic** | **waic** | **cpo** | **pearson_corr** | **spearman_corr** |
| --- | --- | --- | --- | --- | --- | --- | --- |
| model_1_bym_rw1 | 7387720.567 | 9.76E+16 | 8027.523 | 7718.386 | 4897.684 | 0.264603 | 0.931151781 |
| model_1_besag_rw1 | 7518428.694 | 1.01E+17 | 8021.363 | 7738.429 | 4872.225 | 0.264701 | 0.931356559 |
| model_1_iid_rw1 | 7775316.647 | 1.09E+17 | 8026.243 | 7722.252 | 4882.344 | 0.263672 | 0.930989889 |
| model_1_bym_rw2 | 33388.82153 | 1.56E+12 | 16178.64 | 21427.7 | 10895.69 | 0.269046 | 0.834278257 |
| model_1_besag_rw2 | 7022975.563 | 8.75E+16 | 8034.051 | 7810.671 | 4941.257 | 0.266136 | 0.932044637 |
| model_1_iid_rw2 | 7326925.455 | 9.57E+16 | 8026.016 | 7765.863 | 4899.812 | 0.265157 | 0.931608839 |
| model_1_bym_iid | 6371333.649 | 7.14E+16 | 8084.186 | 7970.852 | 5108.761 | 0.267883 | 0.93258091 |
| model_1_besag_iid | 7215972.44 | 9.25E+16 | 8027.126 | 7776.107 | 4907.451 | 0.265845 | 0.931687526 |
| model_1_iid_iid | 7449495.743 | 9.91E+16 | 8025.866 | 7752.408 | 4895.056 | 0.264937 | 0.931530272 |
| model_2_bym_rw1 | 7342043.824 | 9.63E+16 | 8004.995 | 7719.173 | 4808.668 | 0.2646 | 0.931557969 |
| model_2_besag_rw1 | 7341150.177 | 9.62E+16 | 8004.828 | 7719.024 | 4808.266 | 0.264599 | 0.931559443 |
| model_2_iid_rw1 | 7340342.68 | 9.62E+16 | 8004.927 | 7719.247 | 4808.637 | 0.264604 | 0.931559284 |
| model_2_bym_rw2 | 7339860.337 | 9.62E+16 | 8004.836 | 7719.604 | 4808.663 | 0.264615 | 0.931561107 |
| model_2_besag_rw2 | 7346341.711 | 9.64E+16 | 8004.809 | 7719.747 | 4808.066 | 0.264627 | 0.931590353 |
| model_2_iid_rw2 | 7336711.687 | 9.61E+16 | 8004.922 | 7719.345 | 4809.081 | 0.264599 | 0.931568639 |
| model_2_bym_iid | 7334495.493 | 9.61E+16 | 8004.794 | 7719.178 | 4808.681 | 0.264599 | 0.931545296 |
| model_2_besag_iid | 7335150.966 | 9.61E+16 | 8004.961 | 7719.299 | 4809.047 | 0.264598 | 0.931548275 |
| model_2_iid_iid | 7335002.301 | 9.61E+16 | 8004.992 | 7719.372 | 4809.142 | 0.264598 | 0.931550691 |
| model_3_bym_rw1 | 7128677.337 | 9.14E+16 | 8024.622 | 7760.351 | 4897.109 | 0.263567 | 0.931968361 |
| model_3_besag_rw1 | 7267439.508 | 9.50E+16 | 8023.099 | 7744.95 | 4887.017 | 0.263401 | 0.931763434 |
| model_3_iid_rw1 | 7217665.869 | 9.37E+16 | 8023.485 | 7750.566 | 4890.484 | 0.26343 | 0.931804152 |
| model_3_bym_rw2 | 6910891.038 | 8.58E+16 | 8018.777 | 7769.021 | 4901.377 | 0.263727 | 0.931636297 |
| model_3_besag_rw2 | 6849819.311 | 8.37E+16 | 8020.018 | 7764.81 | 4890.353 | 0.265007 | 0.931779041 |
| model_3_iid_rw2 | 7192758.324 | 9.31E+16 | 8023.688 | 7753.149 | 4891.773 | 0.26347 | 0.931876717 |
| model_3_bym_iid | 7167995.348 | 9.24E+16 | 8024.31 | 7756.489 | 4894.812 | 0.263478 | 0.931937082 |
| model_3_besag_iid | 7214469.455 | 9.36E+16 | 8023.337 | 7750.697 | 4889.757 | 0.263431 | 0.931808496 |
| model_3_iid_iid | 7154414.204 | 9.20E+16 | 8024.236 | 7757.363 | 4894.544 | 0.263529 | 0.931963185 |
| model_4_bym_rw1 | 7368949.475 | 9.69E+16 | 8023.98 | 7758.73 | 4890.174 | 0.265061 | 0.931581466 |
| model_4_besag_rw1 | 7367779.339 | 9.69E+16 | 8024.027 | 7758.94 | 4890.404 | 0.265063 | 0.931577352 |
| model_4_iid_rw1 | 7369252.615 | 9.69E+16 | 8023.982 | 7758.697 | 4890.116 | 0.265061 | 0.93157921 |
| model_4_bym_rw2 | 7366883.497 | 9.68E+16 | 8024.03 | 7758.926 | 4890.566 | 0.26505 | 0.931584072 |
| model_4_besag_rw2 | 7369125.881 | 9.69E+16 | 8024.025 | 7758.666 | 4890.374 | 0.265046 | 0.931591952 |
| model_4_iid_rw2 | 7512777.746 | 1.01E+17 | 8022.532 | 7741.647 | 4879.379 | 0.264598 | 0.931402836 |
| model_4_bym_iid | 7369221.273 | 9.69E+16 | 8023.995 | 7758.743 | 4890.078 | 0.265064 | 0.931582627 |

**Table S4.1** Model evaluation model of suicide data for both genders combined.

| model_name | bias | mse | dic | waic | cpo | pearson_corr | spearman_corr |
| --- | --- | --- | --- | --- | --- | --- | --- |
| model_1_bym_rw1 | 14901749 | 5.91E+17 | 7724.802 | 7479.514 | 4757.944 | 0.241637 | 0.931843 |
| model_1_besag_rw1 | 14638921 | 5.73E+17 | 7730.78 | 7510.438 | 4777.148 | 0.241148 | 0.931821 |
| model_1_iid_rw1 | 14603749 | 5.70E+17 | 7728.19 | 7504.235 | 4768.424 | 0.241264 | 0.931861 |
| model_1_bym_rw2 | 14891421 | 5.90E+17 | 7724.393 | 7429.352 | 4757.924 | 0.241628 | 0.931847 |
| model_1_besag_rw2 | 14561011 | 5.67E+17 | 7730.183 | 7512.379 | 4773.307 | 0.24115 | 0.931833 |
| model_1_iid_rw2 | 14523586 | 5.65E+17 | 7729.965 | 7513.385 | 4772.981 | 0.24113 | 0.931849 |
| model_1_bym_iid | 14504290 | 5.63E+17 | 7730.511 | 7514.592 | 4774.446 | 0.241124 | 0.931856 |
| model_1_besag_iid | 13891971 | 5.22E+17 | 7750.232 | 7594.955 | 4825.902 | 0.240139 | 0.931604 |
| model_1_iid_iid | 14483659 | 5.62E+17 | 7730.48 | 7516.148 | 4774.707 | 0.241097 | 0.931863 |
| model_2_bym_rw1 | 14405916 | 5.46E+17 | 7696.071 | 7430.843 | 4663.655 | 0.242714 | 0.931631 |
| model_2_besag_rw1 | 14374117 | 5.42E+17 | 7696.274 | 7430.696 | 4664.719 | 0.242911 | 0.931707 |
| model_2_iid_rw1 | 14456746 | 5.52E+17 | 7696.055 | 7431.282 | 4663.658 | 0.24236 | 0.931605 |
| model_2_bym_rw2 | 14448909 | 5.49E+17 | 7696.363 | 7431.668 | 4663.322 | 0.242635 | 0.931578 |
| model_2_besag_rw2 | 14478101 | 5.51E+17 | 7696.824 | 7433.291 | 4664.44 | 0.242763 | 0.931481 |
| model_2_iid_rw2 | 14484630 | 5.54E+17 | 7696.448 | 7431.236 | 4664.084 | 0.242347 | 0.931581 |
| model_2_bym_iid | 14406997 | 5.46E+17 | 7696.25 | 7430.788 | 4664.031 | 0.242724 | 0.931628 |
| model_2_besag_iid | 14387763 | 5.43E+17 | 7696.242 | 7430.438 | 4664.461 | 0.242918 | 0.93169 |
| model_2_iid_iid | 14460152 | 5.52E+17 | 7696.279 | 7431.044 | 4664.119 | 0.242386 | 0.931598 |
| model_3_bym_rw1 | 13354610 | 4.69E+17 | 7722.36 | 7494.824 | 4767.278 | 0.242654 | 0.931609 |
| model_3_besag_rw1 | 13503407 | 4.81E+17 | 7724.009 | 7495.234 | 4764.548 | 0.242388 | 0.931903 |
| model_3_iid_rw1 | 13275381 | 4.64E+17 | 7723.107 | 7500.843 | 4767.697 | 0.242542 | 0.931575 |
| model_3_bym_rw2 | 13742668 | 4.99E+17 | 7721.932 | 7481.635 | 4759.389 | 0.242308 | 0.931794 |
| model_3_besag_rw2 | 13344374 | 4.68E+17 | 7722.846 | 7495.096 | 4768.693 | 0.242833 | 0.931641 |
| model_3_iid_rw2 | 13352879 | 4.69E+17 | 7721.507 | 7494.132 | 4765.595 | 0.242626 | 0.931594 |
| model_3_bym_iid | 13351141 | 4.69E+17 | 7721.901 | 7494.549 | 4766.969 | 0.24265 | 0.931602 |
| model_3_besag_iid | 13334846 | 4.67E+17 | 7722.715 | 7495.41 | 4768.69 | 0.242842 | 0.931647 |
| model_3_iid_iid | 13354892 | 4.69E+17 | 7721.801 | 7494.184 | 4765.729 | 0.242648 | 0.931607 |
| model_4_bym_rw1 | 14555200 | 5.66E+17 | 7729.263 | 7508.895 | 4772.275 | 0.24121 | 0.93188 |
| model_4_besag_rw1 | 14554011 | 5.66E+17 | 7729.445 | 7509.14 | 4772.404 | 0.241211 | 0.931881 |
| model_4_iid_rw1 | 14551259 | 5.66E+17 | 7729.233 | 7509.228 | 4771.964 | 0.241202 | 0.931876 |
| model_4_bym_rw2 | 14561380 | 5.67E+17 | 7729.352 | 7508.411 | 4772.221 | 0.241214 | 0.931874 |
| model_4_besag_rw2 | 14562196 | 5.67E+17 | 7729.529 | 7508.526 | 4772.278 | 0.241216 | 0.93187 |
| model_4_iid_rw2 | 14557480 | 5.67E+17 | 7729.246 | 7508.449 | 4772.352 | 0.241217 | 0.931878 |
| model_4_bym_iid | 14547272 | 5.66E+17 | 7729.465 | 7509.714 | 4772.826 | 0.2412 | 0.931875 |
| model_4_besag_iid | 14559191 | 5.67E+17 | 7729.387 | 7508.768 | 4772.218 | 0.241213 | 0.93188 |
| model_4_iid_iid | 14558031 | 5.67E+17 | 7729.391 | 7508.856 | 4772.327 | 0.241212 | 0.931886 |

**Table S4.2** Model evaluation model of suicide data for males.

| **model_name** | **bias** | **mse** | **dic** | **waic** | **cpo** | **pearson_corr** | **spearman_corr** |
| --- | --- | --- | --- | --- | --- | --- | --- |
| model_1_bym_rw1 | 7387720.567 | 9.76E+16 | 8027.523 | 7718.386 | 4897.684 | 0.264603 | 0.931151781 |
| model_1_besag_rw1 | 7518428.694 | 1.01E+17 | 8021.363 | 7738.429 | 4872.225 | 0.264701 | 0.931356559 |
| model_1_iid_rw1 | 7775316.647 | 1.09E+17 | 8026.243 | 7722.252 | 4882.344 | 0.263672 | 0.930989889 |
| model_1_bym_rw2 | 33388.82153 | 1.56E+12 | 16178.64 | 21427.7 | 10895.69 | 0.269046 | 0.834278257 |
| model_1_besag_rw2 | 7022975.563 | 8.75E+16 | 8034.051 | 7810.671 | 4941.257 | 0.266136 | 0.932044637 |
| model_1_iid_rw2 | 7326925.455 | 9.57E+16 | 8026.016 | 7765.863 | 4899.812 | 0.265157 | 0.931608839 |
| model_1_bym_iid | 6371333.649 | 7.14E+16 | 8084.186 | 7970.852 | 5108.761 | 0.267883 | 0.93258091 |
| model_1_besag_iid | 7215972.44 | 9.25E+16 | 8027.126 | 7776.107 | 4907.451 | 0.265845 | 0.931687526 |
| model_1_iid_iid | 7449495.743 | 9.91E+16 | 8025.866 | 7752.408 | 4895.056 | 0.264937 | 0.931530272 |
| model_2_bym_rw1 | 7342043.824 | 9.63E+16 | 8004.995 | 7719.173 | 4808.668 | 0.2646 | 0.931557969 |
| model_2_besag_rw1 | 7341150.177 | 9.62E+16 | 8004.828 | 7719.024 | 4808.266 | 0.264599 | 0.931559443 |
| model_2_iid_rw1 | 7340342.68 | 9.62E+16 | 8004.927 | 7719.247 | 4808.637 | 0.264604 | 0.931559284 |
| model_2_bym_rw2 | 7339860.337 | 9.62E+16 | 8004.836 | 7719.604 | 4808.663 | 0.264615 | 0.931561107 |
| model_2_besag_rw2 | 7346341.711 | 9.64E+16 | 8004.809 | 7719.747 | 4808.066 | 0.264627 | 0.931590353 |
| model_2_iid_rw2 | 7336711.687 | 9.61E+16 | 8004.922 | 7719.345 | 4809.081 | 0.264599 | 0.931568639 |
| model_2_bym_iid | 7334495.493 | 9.61E+16 | 8004.794 | 7719.178 | 4808.681 | 0.264599 | 0.931545296 |
| model_2_besag_iid | 7335150.966 | 9.61E+16 | 8004.961 | 7719.299 | 4809.047 | 0.264598 | 0.931548275 |
| model_2_iid_iid | 7335002.301 | 9.61E+16 | 8004.992 | 7719.372 | 4809.142 | 0.264598 | 0.931550691 |
| model_3_bym_rw1 | 7128677.337 | 9.14E+16 | 8024.622 | 7760.351 | 4897.109 | 0.263567 | 0.931968361 |
| model_3_besag_rw1 | 7267439.508 | 9.50E+16 | 8023.099 | 7744.95 | 4887.017 | 0.263401 | 0.931763434 |
| model_3_iid_rw1 | 7217665.869 | 9.37E+16 | 8023.485 | 7750.566 | 4890.484 | 0.26343 | 0.931804152 |
| model_3_bym_rw2 | 6910891.038 | 8.58E+16 | 8018.777 | 7769.021 | 4901.377 | 0.263727 | 0.931636297 |
| model_3_besag_rw2 | 6849819.311 | 8.37E+16 | 8020.018 | 7764.81 | 4890.353 | 0.265007 | 0.931779041 |
| model_3_iid_rw2 | 7192758.324 | 9.31E+16 | 8023.688 | 7753.149 | 4891.773 | 0.26347 | 0.931876717 |
| model_3_bym_iid | 7167995.348 | 9.24E+16 | 8024.31 | 7756.489 | 4894.812 | 0.263478 | 0.931937082 |
| model_3_besag_iid | 7214469.455 | 9.36E+16 | 8023.337 | 7750.697 | 4889.757 | 0.263431 | 0.931808496 |
| model_3_iid_iid | 7154414.204 | 9.20E+16 | 8024.236 | 7757.363 | 4894.544 | 0.263529 | 0.931963185 |
| model_4_bym_rw1 | 7368949.475 | 9.69E+16 | 8023.98 | 7758.73 | 4890.174 | 0.265061 | 0.931581466 |
| model_4_besag_rw1 | 7367779.339 | 9.69E+16 | 8024.027 | 7758.94 | 4890.404 | 0.265063 | 0.931577352 |
| model_4_iid_rw1 | 7369252.615 | 9.69E+16 | 8023.982 | 7758.697 | 4890.116 | 0.265061 | 0.93157921 |
| model_4_bym_rw2 | 7366883.497 | 9.68E+16 | 8024.03 | 7758.926 | 4890.566 | 0.26505 | 0.931584072 |
| model_4_besag_rw2 | 7369125.881 | 9.69E+16 | 8024.025 | 7758.666 | 4890.374 | 0.265046 | 0.931591952 |
| model_4_iid_rw2 | 7512777.746 | 1.01E+17 | 8022.532 | 7741.647 | 4879.379 | 0.264598 | 0.931402836 |
| model_4_bym_iid | 7369221.273 | 9.69E+16 | 8023.995 | 7758.743 | 4890.078 | 0.265064 | 0.931582627 |
| model_4_besag_iid | 7367820.524 | 9.69E+16 | 8024.027 | 7758.912 | 4890.321 | 0.265069 | 0.931582228 |
| model_4_iid_iid | 7370976.639 | 9.69E+16 | 8023.761 | 7758.293 | 4888.902 | 0.26506 | 0.931576684 |

**Table S4.3** Model evaluation model of suicide data for females.

| bias | The expected value of the estimator is not equal to the population parameter. |
| --- | --- |
| mse | The average squared variance between observed and predicted values. |
| dic | The deviance information criterion (DIC) is a hierarchical modeling generalization of the Akaike information criterion (AIC) |
| waic | The widely applicable information criterion (WAIC), also known as Watanabe-Akaike information criterion, is the generalized version of the Akaike information criterion (AIC) onto singular statistical models. |
| cpo | The conditional predictive ordinate (CPO) is a Bayesian diagnostic which detects surprising observations. |
| pearson_corr | Pearson's correlation is a numerical measure of linear relationship between two random variables X and Y. |
| spearman_corr | Spearman's rank correlation is a measure of linear relationship between two ranked variables X and Y. |
| runtime | The run time of the model |
| pdic | pDIC and pWAIC are the effective number of parameters |
| pwaic | pDIC and pWAIC are the effective number of parameters |

**Table S4.4** The column header abbreviations and definition list.

**Supplementary document S5: Maps of hotspots of crude suicide rates in Thailand by gender during the years 1997-2021.**


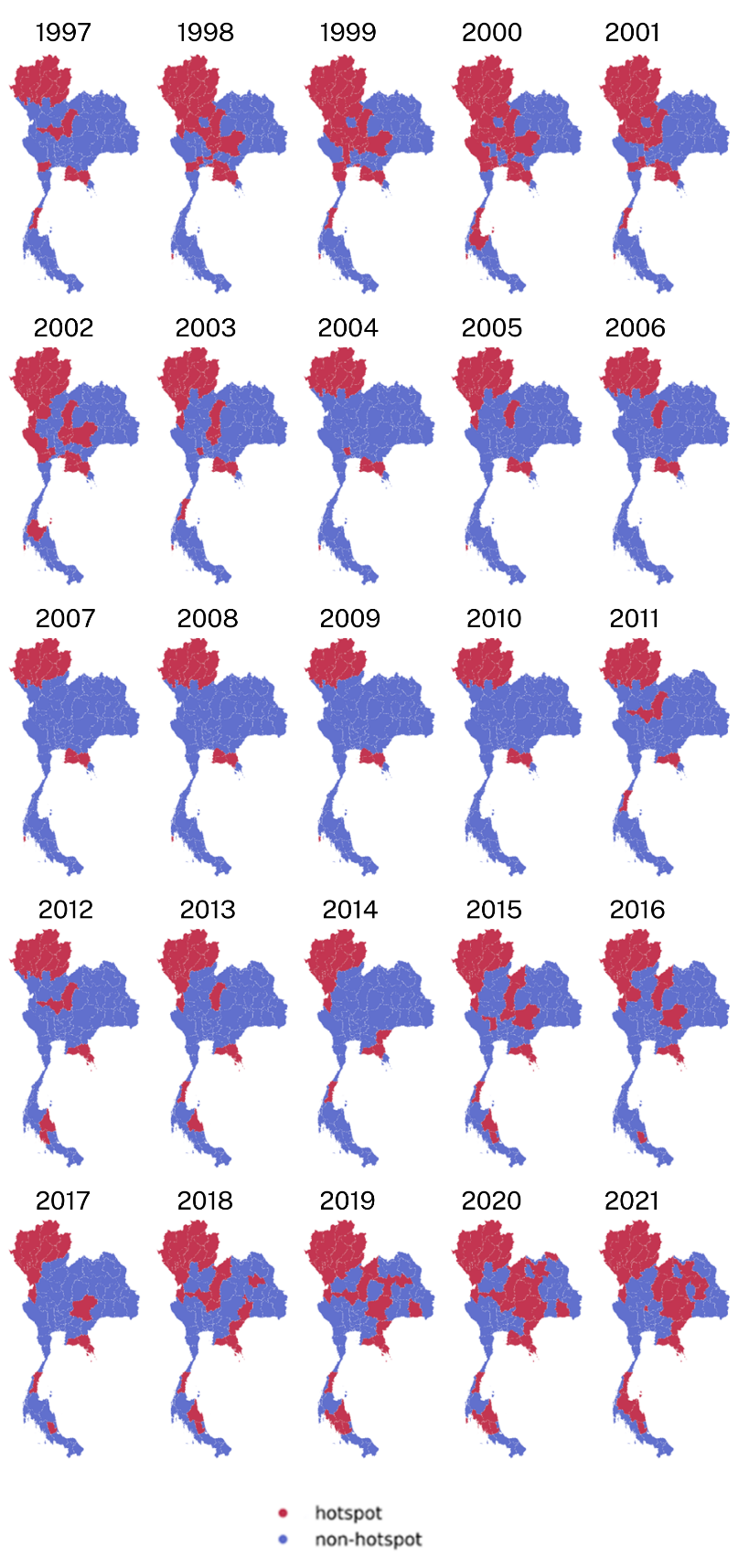


**Figure S5.1** Maps of hotspots of crude suicide rates among males in Thailand during the years 1997-2021, generated using RStudio version 2022.07.0+548 (available at https://posit.co/products/open-source/rstudio/)..


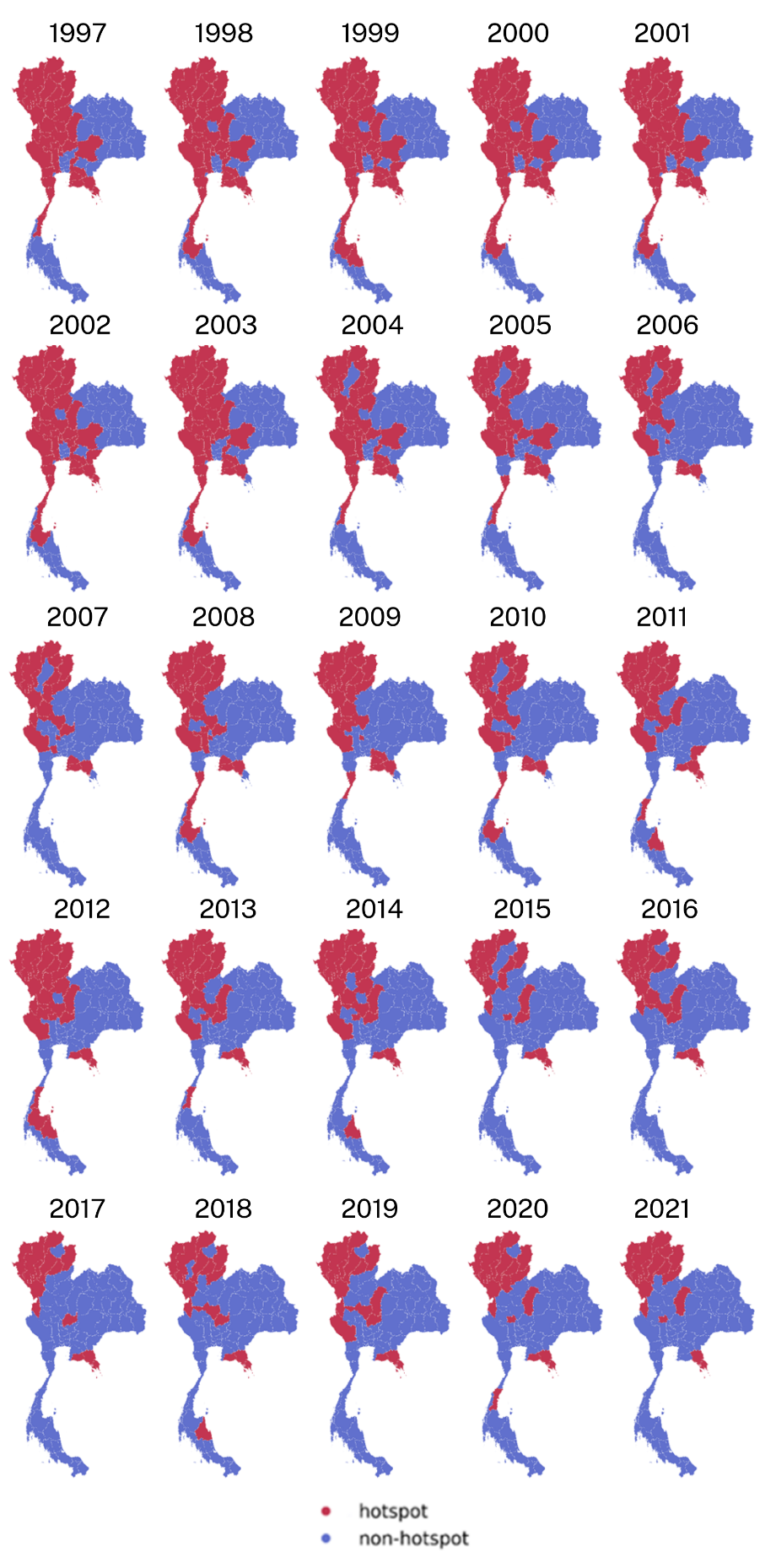


**Figure S5.2** Maps of hotspots of crude suicide rates among females in Thailand during the years 1997-2021, generated using RStudio version 2022.07.0+548 (available at https://posit.co/products/open-source/rstudio/)..

**Supplementary document S6: Maps of association of crude suicide rates and socioeconomic factors in Thailand.**


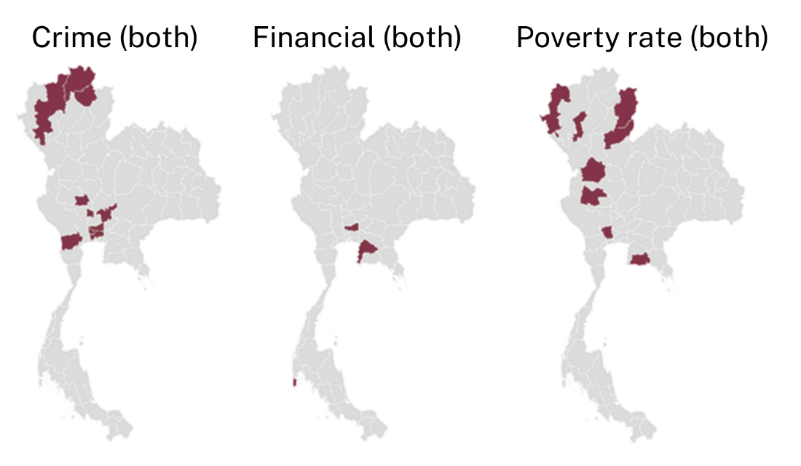


**Figure S6.1** Maps of provincial association with risk factors plot of Thai crude suicide rates both genders combined, generated using RStudio version 2022.07.0+548 (available at https://posit.co/products/open-source/rstudio/).

**References**

1. Besag, J., J. York, and A. Mollié, *Bayesian image restoration, with two applications in spatial statistics.* Annals of the institute of statistical mathematics, 1991. **43**(1): p. 1-20.

2. Knorr‐Held, L., *Bayesian modelling of inseparable space‐time variation in disease risk.* Statistics in medicine, 2000. **19**(17‐18): p. 2555-2567.

3. Rotejanaprasert, C. and A. Lawson, *Bayesian prospective detection of small area health anomalies using Kullback–Leibler divergence.* Statistical methods in medical research, 2018. **27**(4): p. 1076-1087.

4. Rotejanaprasert, C. and A.B. Lawson, *A bayesian quantile modeling for spatiotemporal relative risk: an application to adverse risk detection of respiratory diseases in South Carolina, USA.* International journal of environmental research and public health, 2018. **15**(9): p. 2042.

5. Lawson, A.B., et al., *Handbook of spatial epidemiology*. 2016: CRC press.

6. Aswi, A., et al., *Bayesian spatial and spatio-temporal approaches to modelling dengue fever: a systematic review.* Epidemiology & Infection, 2019. **147**.

7. Lawson, A.B., *Disease cluster detection: a critique and a Bayesian proposal.* Statistics in medicine, 2006. **25**(5): p. 897-916.

8. Lawson, A.B. and C. Rotejanaprasert, *Childhood brain cancer in Florida: a Bayesian clustering approach.* Statistics and Public Policy, 2014. **1**(1): p. 99-107.

9. Rue, H., S. Martino, and N. Chopin, *Approximate Bayesian inference for latent Gaussian models by using integrated nested Laplace approximations.* Journal of the Royal Statistical Society: Series B (Statistical Methodology), 2009. **71**(2): p. 319-392.

10. Chanagul, C., *Determinants of Suicide Rates in Thailand.* Journal of Community Development Research (Humanities and Social Sciences); Vol 12 No 1 (2562): January-March 2019DO - 10.14456/jcdr-hs.2019.2, 2019.

11. Chanagul, C., *Determinants of suicide rates in Thailand.* 2019.

12. Lotrakul, M. and C. Udomswangchoke, *Emerging Trends of Charcoal Burning Suicide in Thailand and Newspaper Reporting.* Journal of the Psychiatric Association of Thailand, 2021. **66**(3): p. 351-364.

13. Rotejanaprasert, C., et al., *Global spatiotemporal analysis of suicide epidemiology and risk factor associations from 2000 to 2019 using Bayesian space time hierarchical modeling.* Scientific Reports, 2025. **15**(1): p. 12785.

14. Schafer, J.L., *Multiple imputation: a primer.* Statistical methods in medical research, 1999. **8**(1): p. 3-15.

15. Dong, Y. and C.-Y.J. Peng, *Principled missing data methods for researchers.* SpringerPlus, 2013. **2**: p. 1-17.

16. Jakobsen, J.C., et al., *When and how should multiple imputation be used for handling missing data in randomised clinical trials–a practical guide with flowcharts.* BMC medical research methodology, 2017. **17**: p. 1-10.

17. Spiegelhalter, D.J., et al., *Bayesian measures of model complexity and fit.* J. R. Stat. Soc. Series B Stat. Methodol., 2002. **64**(4): p. 583-639.

18. Watanabe and Opper, *Asymptotic equivalence of Bayes cross validation and widely applicable information criterion in singular learning theory.* J. Mach. Learn. Res., 2010.

19. Vehtari, A., A. Gelman, and J. Gabry, *Practical Bayesian model evaluation using leave-one-out cross-validation and WAIC.* Statistics and Computing, 2017. **27**(5): p. 1413-1432.
